# Advances in Organic Solvent Nanofiltration Rely on Physical Chemistry and Polymer Chemistry

**DOI:** 10.3389/fchem.2018.00511

**Published:** 2018-10-23

**Authors:** Michele Galizia, Kelly P. Bye

**Affiliations:** School of Chemical, Biological and Materials Engineering, The University of Oklahoma, Norman, OK, United States

**Keywords:** OSN, sorption, diffusion, transport, polymers

## Abstract

The vast majority of industrial chemical synthesis occurs in organic solution. Solute concentration and solvent recovery consume ~50% of the energy required to produce chemicals and pose problems that are as relevant as the synthesis process itself. Separation and purification processes often involve a phase change and, as such, they are highly energy-intensive. However, novel, energy-efficient technologies based on polymer membranes are emerging as a viable alternative to thermal processes. Despite organic solvent nanofiltration (OSN) could revolutionize the chemical, petrochemical, food and pharmaceutical industry, its development is still in its infancy for two reasons: (i) the lack of fundamental knowledge of elemental transport phenomena in OSN membranes, and (ii) the instability of traditional polymer materials in chemically challenging environments. While the latter issue has been partially solved, the former was not addressed at all. Moreover, the few data available about solute and solvent transport in OSN membranes are often interpreted using inappropriate theoretical tools, which contributes to the spread of misleading conclusions in the literature. In this review we provide the state of the art of organic solvent nanofiltration using polymeric membranes. First, theoretical models useful to interpret experimental data are discussed and some misleading conclusions commonly reported in the literature are highlighted. Then, currently available materials are reviewed. Finally, materials that could revolutionize OSN in the future are identified. Among the possible applications of OSN, isomers separation could open a new era in chemical engineering and polymer science in the years to come.

## Introduction

Polymer technology is of paramount importance in several fields. Petrochemical, materials, food and tissue engineering, as well as medicine, optics and microelectronics are some of the areas were polymers endowed with specific structural or functional properties are sought (Haupt and Mosbach, 2000; Ma et al., 2002; Ulbricht, 2006). In this review we discuss the use of functional polymers to achieve molecular separations in liquid phase.

The vast majority of industrial organic synthesis occurs in solution. Downstream processes, such as solute concentration and solvent recovery, play a crucial role in the chemical industry and pose problems that are as relevant as the synthesis process itself (Marchetti et al., 2014). Often, especially in the food or pharmaceutical industry, traditional thermal separation processes, such as distillation, cannot be exploited, since exposure to high temperatures would permanently damage thermally labile molecules, such as drugs, active principles and aromas (Kim et al., 2016; Priske et al., 2016). Liquid chromatography may represent, especially on small scales, an alternative to distillation, however it requires large volumes of solvent to be processed (Liang et al., 2008).

Nowadays, separations consume 15% of the energy produced in the world. Specifically, 80% of the energy demanded to separate chemicals is used for distillation (Lively and Sholl, 2017). Membrane separations operate at relatively mild conditions and do not require a phase change, which makes them less energy demanding relative to thermal processes (Galizia et al., 2017a). Moreover, while distillation requires very large equipment and huge investment costs, membrane modules are compact and much easier to assemble and operate. As a case study, let us consider one cubic meter of acetone solution containing a generic solute. Concentrating by a factor 10 this solution by distillation would require an amount of energy equal to 850 MJ. If the same separation is performed at room temperature and 25 bar using a membrane module, the energy consumption would be 2.2 MJ.

The separations mentioned above are generally classified as organic solvent nanofiltration (OSN) and organic solvent reverse osmosis (OSRO) (Koh et al., 2016; Koros and Zhang, 2017). OSN refers to situations in which a bulky solute, whose molecular mass is in the range 200–1000 g/mol, has to be separated from a solvent. OSN membranes generally operate based on the size sieving mechanism (Marchetti et al., 2014). First, the mixture to be separated is compressed to 30–40 atm and fed to the membrane: bulky solute molecules diffuse slowly through the membrane and, for entropic reasons, tend to be sorbed to a small extent by the membrane materials so, based on the solution-diffusion model (cf. section Solution-Diffusion model), they are rejected by the membrane and concentrated in the retentate side. Conversely, small solvent molecules preferentially permeate through the membrane (Marchetti et al., 2014). During the process, the downstream side of the membrane is kept at atmospheric pressure, so the pressure difference across the membrane drives the selective permeation of the solvent over the solute. OSRO refers to membrane separation of two or more liquids of like size. Since the currently available polymer materials cannot accomplish the latter separation, OSRO research efforts are being directed toward inorganic membranes based on carbon molecular sieves (Koros and Zhang, 2017; Lively and Sholl, 2017). Such materials separate molecules of like size based on the so-called entropic selectivity (Singh and Koros, 1996; Koros and Zhang, 2017).

There are several incentives for using OSN in the industrial practice. First, membrane filtration can be used either alone or in combination with traditional processes, such as distillation or liquid chromatography, giving rise to the so-called process intensification. As demonstrated by several techno-economic studies, such practice would reduce significantly the energy consumption of traditional processes, while ensuring high separation efficiency (White, 2006; Van der Bruggen, 2013; Marchetti et al., 2014; Szekely et al., 2014). Another advantage for using membrane filtration is its flexibility. Indeed, three operating modes are possible for OSN membranes, namely concentration, purification, and solvent exchange. Concentration processes involve the separation of a single solute from a solvent. As mentioned above, solute is concentrated in the retentate side, and solvent is recovered in the permeate side. Purification processes consist in separating two or more solutes, for example the main product of a chemical reaction from the byproduct. Finally, solvent exchange is used to enrich a solution in the solvent B by removing the solvent A.

Although ceramic materials have been also explored, polymers appear more suitable for OSN applications. The large amount of energy required to fabricate defect-free ceramic membranes, as well their intrinsic brittleness, represent the most relevant drawbacks for using ceramics in membrane manufacturing (Li, 2007).

Although OSN membranes are supposed to work based on the size exclusion mechanism, several factors, such as chemical-physical properties of the solute, solute-membrane interactions, solvent-membrane interactions, and membrane structure influence the membrane performance (Tsarkov et al., 2012; Postel et al., 2013; Volkov et al., 2014, 2015; Ben Soltane et al., 2016; Marchetti et al., 2017). However, little research efforts have been devoted to the analysis of these fundamental aspects.

The first attempt to use polymer membranes for organic liquid separation dates back to 1964, when Sourirajan used cellulose acetate to separate xylene from ethanol (Sourirajan, 1964). For almost 30 years, cellulose acetate was considered the standard material for separations involving organic liquids (Kopecek, 1970; Fang et al., 1992). The lack of materials capable to withstand chemically challenging environments has represented, for a long time, the real roadblock to progress in OSN (Cook et al., 2018). Indeed, organic solvents are sorbed to a significant extent by glassy and rubbery polymers. Such very high sorption, which often exceeds 50% by weight, may prejudice the membrane stability. Sometime, polymers can even dissolve in the presence of organic solvents. Several stable materials were developed in the last two decades, so the issue of membrane stability can be considered partially solved. Among these materials, cross-linked Lenzing P84 polyimide (White, 2001, 2002) has been particularly successful and it made possible small scale industrial applications of OSN in dewaxing of lube oil, homogeneous catalyst recovery, and enrichment of aromatics.

In the last decade, research has been focusing on tough polymers. This class of materials includes glassy polymers made of condensed aromatic rings with superior stability in solvents, such as polybenzimidazoles (Vogel and Marvel, 1961; Valtcheva et al., 2014, 2015; Borjigin et al., 2015) and polymers of intrinsic microporosity (PIMs) (Budd et al., 2004; Fritsch et al., 2012; Gorgojo et al., 2014; Ogieglo et al., 2017). Perfluorinated polymers (Chau et al., 2018), polyether ether ketone (PEEK) (Da Silva Burgal et al., 2015, 2017), and polyarylates (Jimenez-Salomon et al., 2016) also received significant attention for separations in harsh environments. In this review, we provide a detailed discussion of these materials, including synthesis routes, transport properties and property-structure correlations.

New interesting applications of OSN are in the pharmaceutical industry and, more specifically, in the downstream processing of drugs, peptides and oligonucleotides. Noteworthy, membrane filtration in pharmaceutical industry can be exploited for separations, extractions, drying and also for particles formation (i.e., crystallization) (Buonomenna and Bae, 2015). For example, oligonucleotides and peptides are synthesized in solution and require a thorough purification before use. OSN represents a valid alternative to other processes, such as distillation or solvent extraction, which could potentially damage thermally and chemically labile biomolecules. Membranes based on cross-linked polybenzimidazole were successfully used to purify peptides and oligonucleotides, opening a new avenue in the area of oligo therapeutics (Kim et al., 2016).

Although membrane separation of organics has been indicated as one of the seven molecular separations that could change the world in the next decades (Lively and Sholl, 2017), OSN is currently one of the most poorly understood processes at a fundamental level. Specifically, the lack of fundamental knowledge about molecular factors governing solute and solvent transport in OSN membranes hampers OSN to take off the ground. Fundamental understanding of transport phenomena in OSN membranes would help not only to develop guidelines for the rational design of materials with pre-assigned transport properties, but also to select the optimal operative conditions to maximize the productivity and selectivity of existing membranes. Such aspect constitutes an essential prerequisite to make OSN competitive with traditional processes.

In this review we provide the state of the art of OSN using polymeric membranes. First, theoretical models useful to interpret experimental data are discussed and some misleading conclusions commonly reported in the literature are highlighted. The correct interpretation of OSN data is an essential pre-requisite to identify the key factors affecting solute and solvent transport in OSN membranes. Specifically, the individuation of a general framework to interpret transport data in OSN membranes represents the first step toward the development of structure-property correlations useful for the rational design of OSN membranes. A general theoretical framework for OSN is presented in section Transport Phenomena is OSN Membranes: Mechanism and Challenges. Then, currently available materials are reviewed. Finally, materials that could revolutionize OSN in the future are identified. For each of them, synthesis routes and structure-property correlations are presented and discussed.

## Transport phenomena is OSN membranes: mechanism and challenges

### Solution-diffusion model

The solution-diffusion model sets the mechanism by which gases, vapors, liquids, and ions diffuse through dense (i.e., non-porous) polymers (Paul, 1973, 2004; Wijmans and Baker, 1995). Leveraging on an elegant series of experiments and a purposely built theory, Paul and coworkers, in the early 70's, demonstrated that the solution-diffusion mechanism rules the pressure driven transport of organic liquids in rubbery polymers (Paul and Ebra-Lima, 1971). The pioneering studies conducted by Paul can be considered the first attempt to understand OSN at a fundamental level, although, at the time, OSN was not recognized as such.

**Figure 1:**
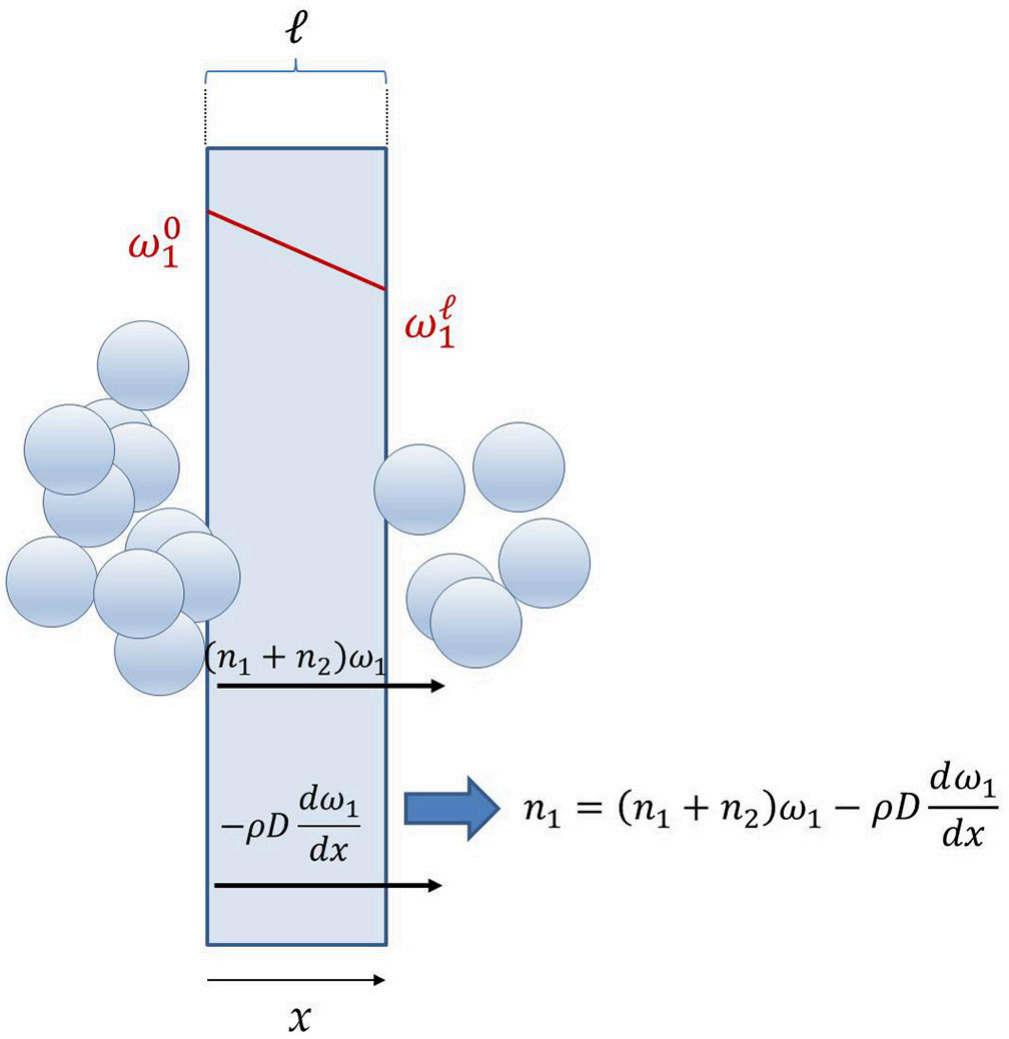
Schematic of small molecule transport through dense polymer membranes.

According to the solution-diffusion model, penetrant molecules first partition between the feed and the upstream face of the membrane. Then, sorbed molecules diffuse through the membrane thickness, down a concentration (or chemical potential) gradient. Finally, they desorb from the downstream face of the membrane. When considering the transport of liquids through dense polymer membranes, the permeability coefficient, which is an intrinsic property of the membrane material, is defined as the pressure and thickness normalized flux (Paul, 1973):

(1) $$P_{1}=\frac{n_{1}l}{\rho_{1}\Delta p}\;$$

where subscripts 1 and 2 stand for the penetrant and the membrane species, respectively. In Equation 1, *n*_1_ is the steady-state penetrant mass flux relative to the fixed frame reference, i.e., the membrane, ρ_1_ is the penetrant density, Δ*p* is the pressure difference across the membrane, and ***l*** is the membrane thickness. To simplify the matter, let us consider the diffusion of a pure liquid through a polymer. To quantitatively describe this phenomenon, it is necessary to define a frame of reference. Two frames of reference can be used, namely fixed or moving (Paul, 1973; Kamaruddin and Koros, 1997; Galizia et al., 2016). In the moving framework, the diffusional flux is defined with respect to the center of mass of the polymer-penetrant system, which, in turn, moves with its own bulk velocity. Within this framework, the diffusional velocity relative to the bulk velocity causes the penetrant flux through the membrane. In the fixed or stationary frame of reference, the membrane itself is used as the frame of reference. The penetrant mass flux through the membrane, *n*_1_, with respect to the fixed frame is expressed as follows (Paul, 1973; Kamaruddin and Koros, 1997; Galizia et al., 2016):

(2) $$n_{1}=\;n_{1}^{diff}+n_{1}^{conv}=j_{1}+\omega_{1}\left(\;n_{1}+n_{2}\right)$$

where *j*_1_ is the diffusive mass flux, i.e., the mass flux of species 1 relative to the center of mass of the polymer-penetrant system, ω_1_ is the penetrant mass fraction in the membrane, and *n*_2_ is the membrane flux (cf. Figure 1). This result can be extended to multicomponent permeation, so that, with respect to the fixed frame of reference, the mass flux of each permeating species is given by the convective flux resulting from the bulk motion of the permeating mixture (second term at the right-hand side in Equation 2), plus the diffusive flux (first term at the right-hand side in Equation 2).

In case of Fickian diffusion, *j*_1_ is expressed as follows:

(3) $$j_{1}=-\rho D_{1}\frac{d\omega_{1}}{dx}$$

where *D*_1_ is the effective, concentration averaged penetrant diffusion coefficient through the membrane, ρ is the density of polymer-penetrant mixture, and *x* is the generic abscissa through the membrane thickness. Combining Equations 2, 3 and remembering that at steady state the polymer flux, *n*_2_, is zero, leads to the following expression for the penetrant mass flu with respect to the fixed frame of reference:

(4) $$n_{1}=-\left(\frac{\rho D_{1}}{1-\omega_{1}}\right)\frac{d\omega_{1}}{dx}$$

During a permeation experiment, penetrant flux is measured with respect to the membrane, i.e., the experimentally measured flux is *n*_1_ (Paul, 1973). However, when considering gas and vapor transport in polymers, ω_1_ is vanishing, so that 1−ω_1_≅1. In this situation, the experimentally measured flux, *n*_1_, is essentially equal to the diffusive flux, *j*_1_. In contrast, liquid solvent uptake by polymers may be significant, so 1−ω_1_≪1, which means that *n*_1_ significantly departs from *j*_1_. In the latter situation, neglecting the factor 1/(1−ω_1_) in Equation 4 would cause significant errors in the estimation of the diffusion coefficient (Paul, 1973).

As the approach presented above can be generalized to the diffusion of multicomponent mixtures, Equation 4 should be used to describe solvent and solute transport in OSN membranes. Interestingly, such approach can be further generalized to account for the effects of osmotic pressure and concentration polarization (Peeva et al., 2004). Details about integration of Equation 4 were reported by Dinh (Dinh et al., 1992) and Paul (Paul, 1973). Integration of Equation 4 requires that the penetrant concentration profile in the membrane is known. As reported in previous studies (Paul, 2004; Galizia et al., 2016), the ω_1_ profile can be considered linear, so that $\omega_{1}=\omega_{1}^{0}\left(1-\frac{x}{l}\right)$, where $\omega_{1}^{0}$ is the penetrant concentration in the upstream face of the membrane, which can be estimated from liquid sorption experiments.

Correcting the Fick's law for the frame of reference is not sufficient. When considering a membrane separating two solutions with different compositions, the chemical potential gradient across the membrane is the driving force for penetrant diffusion (Bird et al., 1960). However, as concentration is much easier to measure than chemical potential, often the concentration gradient across the membrane is assumed as the actual driving force for penetrant transport. Indeed, in writing Equation 4, we followed the latter approach. When the driving force is expressed in terms of concentration gradient, the diffusion coefficient appearing in Equation 4, *D*_1_, is the product of a purely kinetic quantity, i.e., the mobility coefficient, *L*_1_ , which accounts for the frictional resistance offered by the polymer matrix to penetrant diffusion, and the thermodynamic factor, $\frac{1}{RT}\frac{\partial \mu_{1}}{\partial ln\omega_{1}}$ , related to polymer-penetrant interactions (Doghieri and Sarti, 1997; Galizia et al., 2011):

(5) $$D_{1}=L_{1}\left(\frac{1}{RT}\frac{d\mu_{1}}{d\ln\omega_{1}}\right)$$

In Equation 5, μ_1_ is the penetrant chemical potential in the polymer-penetrant mixture and ω_1_ is the penetrant mass fraction in the polymer. When polymer-penetrant mixing is ideal (i.e., no special interactions take place), the factor $\left(\frac{1}{RT}\frac{d\mu_{1}}{dln\omega_{1}}\right)$ is equal to one. Conversely, when polymer-penetrant mixing is non-ideal, which happens frequently, the thermodynamic factor deviates from one. Specifically, when the polymer and penetrant exhibit like physical-chemical properties and their mutual interactions are thermodynamically favorable, $\left(\frac{1}{RT}\frac{d\mu_{1}}{dln\omega_{1}}\right)>1$. Conversely, when polymer-penetrant interactions are thermodynamically unfavorable, $\left(\frac{1}{RT}\frac{d\mu_{1}}{dln\omega_{1}}\right)<1$ (Doghieri and Sarti, 1997; Galizia et al., 2011). This aspect complicates significantly the analysis of diffusion coefficients, which need to be corrected also for thermodynamic non-idealities. While the latter issue has been addressed by Paul for rubbery membranes, based on the Flory-Huggins theory (Paul, 1973), no general solution has been proposed so far for glassy membranes. Such aspect represents a potential research topic *per se*, since polymers used for OSN are typically glassy. A very simple method to correct diffusion coefficients in glassy polymer for thermodynamic non-idealities relies on vapor sorption data. Gas and vapor sorption isotherms in glassy polymers can be described using the dual mode sorption model (Koros et al., 1976), which provides the following analytic expression for the thermodynamic factor (Merkel et al., 2000):

(6) $$\frac{1}{RT}\frac{d\mu_{1}}{d1n\omega_{1}}=\frac{k_{D}+\frac{C^{\prime }H^{b}}{1+bp}}{k_{D}+\frac{C^{\prime }H^{b}}{{\left(1+bp\right)}^{2}}}\left[1+\frac{M_{w}}{22414\rho_{p}}\left(k_{D}p+\frac{C^{\prime }H^{b}p}{1+bp}\right)\;\right]$$

where $k_{D},\;\;CH^{\prime }$ and *b* are the three dual mode parameters, which can be estimated by fitting vapor sorption isotherms, *p* is the vapor partial pressure, *M*_*w*_ is the penetrant molecular mass and ρ_*p*_ is the polymer density. If vapor sorption data are available, the thermodynamic factor can be calculated as a function of activity using Equation 6, and then it can be extrapolated at activity 1, i.e., to the case of liquid sorption. However, such very simple method does not always work. For example, lower alcohols (e.g., methanol and ethanol) sorption is some glassy polymers, such as polyacetylenes, cannot be described by the dual mode model, due to the occurrence of significant penetrant clustering (Galizia et al., 2011, 2012). For this reason, advanced methods based on more fundamental models are under development. For example, the non-equilibrium lattice fluid model provides a general, analytic expression for the penetrant chemical potential in mixture with a glassy polymer (Doghieri and Sarti, 1996), from which the derivative appearing in Equation 5 can be calculated analytically.

For rubbery polymers, the diffusion coefficient corrected for the frame of reference and thermodynamic non-idealities is given by (Paul, 1973):

(7) $$L_{1}=D_{1}{\left(1-\phi_{1}\right)}^{2}\left(1-2\chi_{1}\phi_{1}\right)$$

where *D*_1_ is the experimentally determined diffusion coefficient, ϕ_1_ is the penetrant volume fraction in the membrane, and χ_1_ is the polymer-penetrant Flory-Huggins interaction parameter (Flory, 1942).

Paul (1973) successfully used this approach to describe a set of experimental diffusion data presented by White (1960). White measured pure liquid water transport through rubbery polyacrylamide membranes whose water content was about 70% vol. Interestingly, water diffusion coefficient through the membrane, calculated as the ratio of permeability to sorption coefficients, resulted one order of magnitude larger than water self-diffusion coefficient (i.e., 2.8 × 10^−5^ cm^2^/s). To explain this unexpected result, White speculated that, for such highly swollen membrane, water transport occurs by pore flow. Paul demonstrated that the results presented by White could be rationalized using Equations 4 and 7 (Paul, 1973). Indeed, since the polyacrylamide membrane was highly swollen, neglecting the term 1/(1−ω_1_) in Equation 4, as well as thermodynamic non-idealities, led White to overestimate the diffusion coefficient by orders of magnitude. After correction for the effect of the frame of reference and thermodynamic non-ideality, water diffusion coefficients in polyacrylamide were far below the liquid water self-diffusion coefficient. So, invoking pore flow to describe water transport in polyacrylamide membranes was inappropriate.

In recent years, similar conclusions were drawn by Volkov et al. (1996) when assessing the suitability of glassy poly(trimethylsilyl propyne) (PTMSP) for OSN application. They observed that liquid ethanol diffusion coefficient in PTMSP (i.e., 1.27 × 10^−8^ cm^2^/s) was larger than ethanol self-diffusion coefficient (i.e., 1.10 × 10^−9^ cm^2^/s) and speculated occurrence of pore flow transport. This conclusion is dramatically altered if the experimental data are analyzed using the Paul's approach. From swelling experiments, ethanol volume fraction in PTMSP at room temperature resulted 0.46. If one assumes, in first approximation, the Flory-Huggins theory to account for thermodynamic non-ideality, the χ parameter can be estimated from liquid ethanol sorption data. Doing so, gives χ = 0.813. Finally, from Equation 7, the ethanol diffusion coefficient in PTMSP corrected for the frame of reference and thermodynamic non-idealities is 9.30 × 10^−10^ cm^2^/s, which is below the ethanol self-diffusion coefficient and fifteen times smaller relative to the value calculated by Volkov. The dual mode approach, although more appropriate for glassy PTMSP, cannot be used to correct ethanol diffusion coefficient for thermodynamic non-idealities, since ethanol sorption is PTMSP is not dual-mode type (Doghieri and Sarti, 1997).

The examples discussed above show how poor has been, so far, the fundamental analysis of transport phenomena in OSN membranes, and how this issue continues to generate misleading interpretations of experimental data.

The solution-diffusion model links penetrant permeability, solubility and diffusion coefficients in the membrane as follows (Wijmans and Baker, 1995):

(8) $$P_{1}=D_{1}\times S_{1}$$

**Figure 2:**
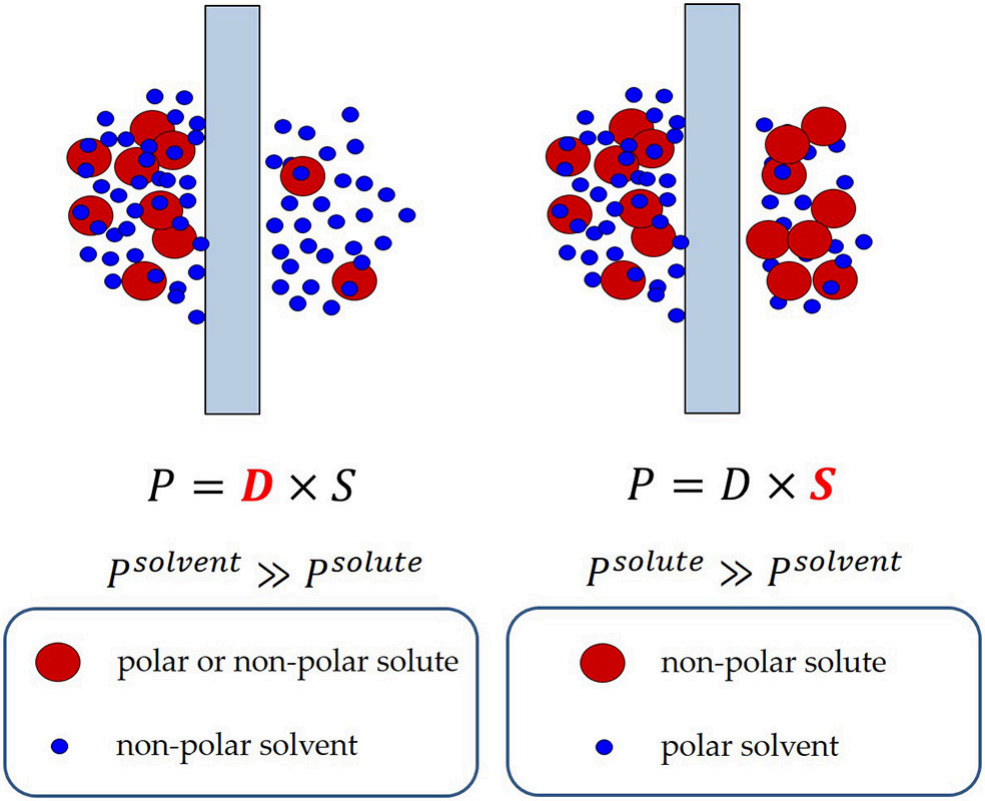
Solvent and solute transport in PDMS-based membranes. On the right is reported the case where solute permeability exceeds solvent permeability. Solute preferential sorption is believed to be the cause of this behavior (Postel et al., 2013).

When considering the permeation of a binary mixture through a membrane, the selectivity for the component *i* vs. the component *j* is defined as follows (Wijmans and Baker, 1995):

(9) $$\alpha_{ij}=\frac{P_{i}}{P_{j}}=\frac{D_{i}}{D_{j}}\times \frac{S_{i}}{S_{j}}=\alpha_{D}\times \alpha_{S}$$

where α_*D*_ and α_*S*_ are the diffusivity-selectivity and the solubility-selectivity, respectively. The diffusivity-selectivity is believed to control the selective permeation of solvent over solute through OSN membranes. Indeed, since diffusivity decreases markedly with increasing penetrant size, solute molecules are expected to be rejected by the membrane due to their very slow diffusion in the membrane material. However, deviations from this rule of thumb have been observed. For example, Postel reported that during removal of non-polar hydrocarbon solutes (e.g., alkanes) from polar solvents (e.g., isopropanol) using rubbery PDMS membranes, solute permeability through the membrane largely exceeds solvent permeability (Postel et al., 2013). As a consequence, the solute is enriched in the permeate side relative to the feed side (i.e., the membrane is solute-selective instead of solvent-selective, cf. Figure 2). Postel hypothesized that preferential solute sorption in PDMS, caused by its higher thermodynamic affinity with the polymer, is responsible for this phenomenon, even though no direct proof of this hypothesis has been provided (Postel et al., 2013).

As we will see in the forthcoming sections, often commercial membranes are made by a thin active layer, which actually performs the separation, cast onto a porous support. Since the thickness of the active layer cannot be measured accurately, application of Equation 1 to calculate permeability is not straightforward. So, the performance of these composite membranes is expressed in terms of permeance instead permeability. Permeance is the thickness normalized permeability and, as such, it is not an intrinsic property of the membrane, since it depends on its geometry. Likewise, in practical applications, rejection is used instead selectivity. Rejection of species *i* is defined as follows (Marchetti et al., 2014, 2017):

(10) $$R_{i}=1-\frac{C_{i}^{p}}{C_{i}^{f}}$$

where $C_{i}^{p}$ and $C_{i}^{f}$ are the concentrations of species *i* in the permeate and feed, respectively. Rejection is related to selectivity as follows:

(11) $$\alpha_{ij}=\frac{1-R_{i}}{1-R_{j}}$$

### Plasticization

Membrane plasticization indicates an increase in chain mobility caused by penetrant sorption (Chiou et al., 1985). Such improved chain mobility reduces the energy penalty required to open gaps between polymer chains, thus enhancing penetrant diffusivity and permeability. However, such an increase in permeability is accompanied by a loss in membrane selectivity. OSN membranes must exhibit adequate resistance to plasticization, as they face directly organic liquids during their operation. Methods to mitigate membrane plasticization rely essentially on polymer chemistry: covalent or ionic cross-linking, as well as use of rigid monomers with limited rotation freedom are some relevant examples (Vanherck et al., 2013; Valtcheva et al., 2014, 2015).

### Physical aging

Rigid materials, such as glassy polymers, are sought in OSN applications. Glassy polymers contain an excess free volume trapped in their structure and, as such, they are non-equilibrium materials. Excess free volume is *per se* unstable and tends to be relaxed over time, producing a progressive densification of the polymer matrix. This process is referred to as physical aging (Huang and Paul, 2004, 2006). Loss of free volume significantly affects transport properties of glassy membranes, as it reduces small molecule permeability and enhances selectivity. Paul and co-workers demonstrated that the physical aging rate of glassy polymer films is inversely proportional to their square thickness, which justifies the accelerated aging exhibited by thin films typically used in membrane applications (Huang and Paul, 2004, 2006). Membranes 1 μm thick or less lose 25% of their permeability within a few days after manufacturing. Another 25% of their permeability is lost in the following 15 days (Galizia et al., 2017a). For example, asymmetric membranes based on cross-linked Lenzing P84 polyimide, the current standard material for OSN, exhibit solvent permeance ranging from 1 to 2 L/(m^2^ h bar). Thermal annealing at 200°C decreases solvent permeance to zero, due to polymer densification (See See-Toh et al., 2007a). The issue of physical aging becomes dramatic during operation at medium-high temperatures, as polymer chains motion, who is ultimately responsible for physical aging of glassy polymers, becomes faster with increasing temperature.

Strategies to mitigate this issue have been devised, which rely on covalent cross-linking and use of rigid monomers with frustrated rotation freedom. While the former strategy was sometimes successful, the latter did not always guarantee good results, as rigid polymers, such as PIMs (Bernardo et al., 2017) and thermally rearranged (TR) polymers (Wang et al., 2014) also exhibit physical aging when manufactured as thin films.

Recently, Livingston and co-workers reported negligible aging in poly(ether ether ketone) (PEEK) membranes for OSN (Da Silva Burgal et al., 2015, 2017). PEEK is a fully aromatic, semi-crystalline polymer made by hydroquinone and benzophenone segments. Physical aging of bulky membranes (10–50 μm) typically used in the academia is a slow process and its effects do not show up within experimentally accessible timescales (Galizia et al., 2017a). To accelerate physical aging, Da Silva Burgal annealed thick films made by PEEK, Lenzing P84 polyimide and polybenzimidazole at 120°C for 48 h (Da Silva Burgal et al., 2017). Polyimide and PBI samples became very brittle after thermal annealing, due to the accelerated free volume relaxation, which reduced polymer chain mobility. Their Young's modulus could not be measured after annealing. In contrast, PEEK didn't show any brittleness and its Young's modulus increased from 61 MPa to 108 MPa after annealing at 120°C. The unusual stability exhibited by PEEK is likely due to the presence of ultra-rigid crystalline domains, which freeze polymer chains in the amorphous domain in their original position (Da Silva Burgal et al., 2017).

**Figure 3:**
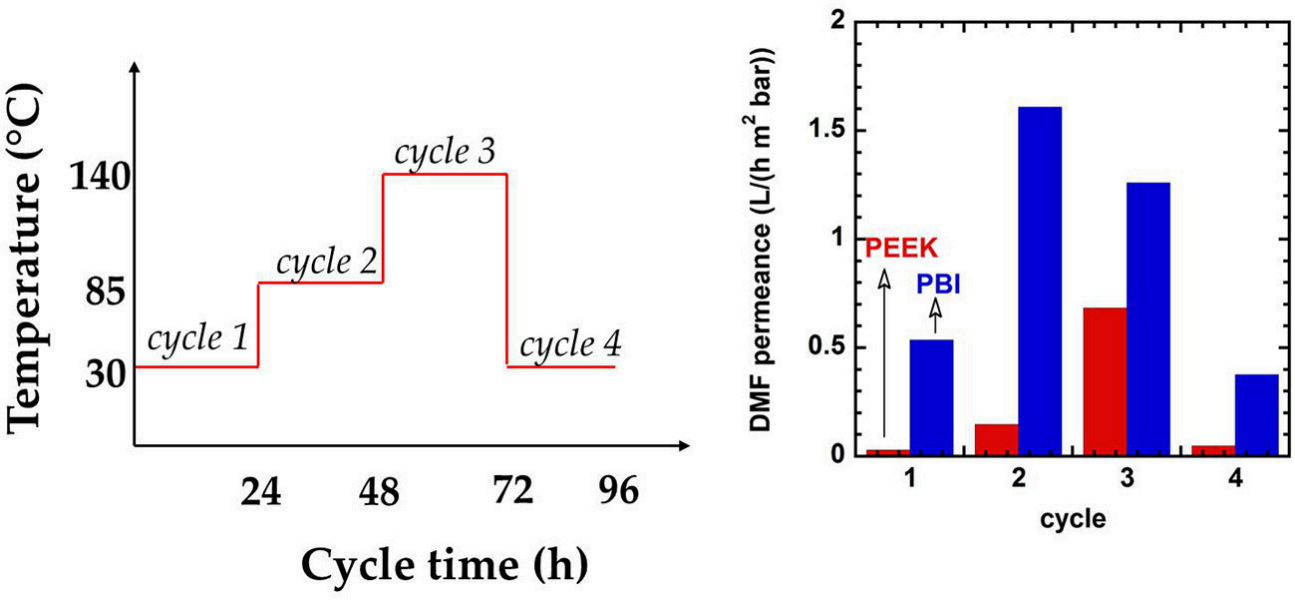
Effect of physical aging on poly (ether ether ketone) and polybenzimidazole membranes. Adapted from Da Silva Burgal et al. (2017), with permission of *Elsevier*.

Three dimethylformamide (DMF) filtration experiments were run with PEEK, polyimide P84 and PBI membranes at 30, 85, and 140°C, respectively, followed by a fourth run at 30°C. Permeance of P84 decreased by a factor 6 from 30 to 85°C. At 140°C, cross-linking thermal scission caused the membrane failure. PEEK and PBI permeance increased by a factor 20 and 2.5, respectively, from 30 to 140°C. Interestingly, during the fourth filtration cycle, when temperature was returned to 30°C, PEEK exhibited the same permeance as in the first run, and PBI lost 25% of its original permeance (cf. Figure 3). This result was justified by invoking negligible aging in PEEK (Da Silva Burgal et al., 2017).

### Permeability-selectivity trade-off

**Figure 4:**
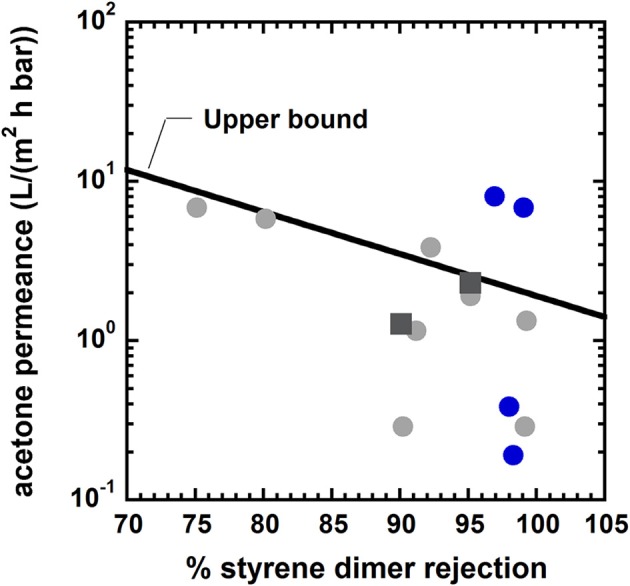
Upper bound diagram for styrene dimers (molecular mass 236 g/mol) separation from acetone at room temperature. Solid gray circles: conventional asymmetric membranes. Solid black square: conventional composite membranes. Solid blue circles: nanoporous polyarylates synthesized by Jimenez-Salomon et al. (2016). Blue circles above the upper bound refer to polyarylates obtained using contorted, non-planar monomers [i.e., 9,9-bis(4-hydroxyphenyl)fluorine]. Blue circles below the upper bound refer to polyarylates obtained using planar, non-contorted monomers (i.e., dihydroxyanthraquinone). Adapted from Jimenez-Salomon et al. (2016), with permission of *NatureResearch*.

The existence of a trade-off between permeability and selectivity in polymer membranes for gas separation was reported on empirical bases by Robeson (Robeson, 1991, 2008), and it was explained theoretically by Freeman (1999). Generally, highly permeable membranes exhibit poor selectivity, and vice-versa. As a consequence, the performance of polymer membranes is limited to stay below the upper bound defined by the best performing materials. Conversely, inorganic materials, such as zeolites, often surpass the upper bound, as they exhibit high levels of permeability and selectivity. Recently, a permeability-selectivity trade-off for OSN membranes was reported by Marchetti (Marchetti et al., 2017). Such finding strongly supports the idea that the solution-diffusion mechanism rules solute and solvent transport in OSN membranes. An example of permeance/rejection upper bound for OSN is reported in Figure 4.

### Effect of pressure on solvent flux

**Figure 5:**
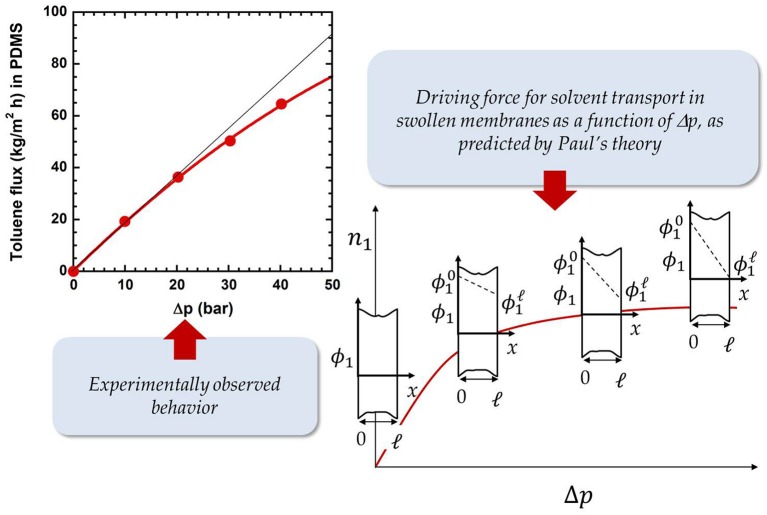
Toluene flux through PDMS as a function of Δp at room temperature. Data were taken from Postel et al. (2013). The evolution of driving force for solvent transport as a function of Δp is reported as well, adapted from Paul (2004), with permission of *Elsevier*.

One of the most controversial aspects in OSN is the dependence of solvent flux on the upstream pressure. Several authors observed that flux is linear with Δp (i.e., the pressure difference across the membrane), and it declines at high pressures. Membrane compaction caused by the upstream pressure has been invoked to explain this behavior (Machado et al., 1999; Grekhov et al., 2012; Ben Soltane et al., 2013). However, while this explanation could be physically sound for soft, rubbery membranes, such as PDMS (elastic modulus < 4 MPa), it seems unrealistic for rigid glassy polymers typically used in OSN (elastic modulus ≅ 4 GPa), especially if we consider that flux decline often starts at relatively low pressures, such as 10–12 bar. We believe the right explanation of this phenomenon should be found in the pioneering studies from Paul and co-workers (Paul and Ebra-Lima, 1970, 1971; Paul, 1973). Paul demonstrated that the driving force for liquid transport through a swollen membrane is the swelling gradient between the upstream and the downstream face of the membrane, $\phi_{1}^{0}-\phi_{1}^{l}$ , where ϕ is the liquid volume fraction in the membrane material (Paul, 1973). While $\phi_{1}^{0}$ (i.e., the liquid volume fraction in the upstream face) is constant, as it does not depend on the applied pressure, $\phi_{1}^{l}$ (i.e., the liquid volume fraction in the downstream face) decreases with increasing upstream pressure. When the upstream pressure is high enough, $\phi_{1}^{l}$ can become zero. In this situation, the maximum driving force is available, which means that flux cannot further increase with increasing pressure (cf. Figure 5). Paul derived the following expression for solvent mass flux as a function of applied Δ*p* (Paul, 1976, 2004):

(12) $$n_{1}=\frac{C_{1}^{0}D_{1}}{l\left(1-\omega_{1}^{0}\right)}\left[1-\;\exp\left(-\frac{{\tilde{V}}_{1}\Delta p}{RT}\right)\right]$$

where $C_{1}^{0}$ and $\omega_{1}^{0}$ are the solvent concentration and mass fraction at the upstream face of the membrane, respectively, Ṽ_1_ is the solvent molar volume and Δ*p* is the pressure difference across the membrane. Equation 12 states that the ceiling flux is attained more rapidly with increasing permeant molar volume.

**Figure 6:**
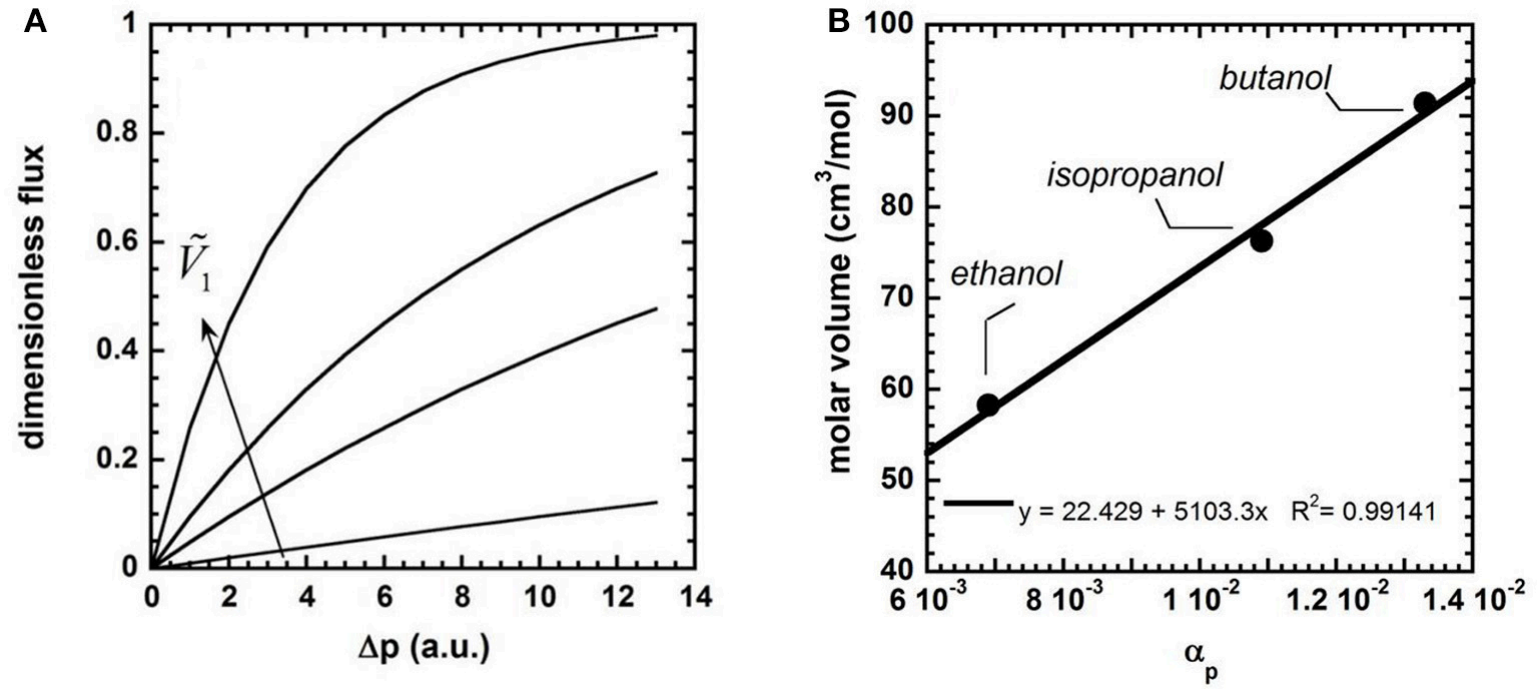
**(A)** Solvent dimensionless flux vs. Δp at various solvent molar volumes, as predicted by the Paul theory (Paul, 1973). **(B)** Solvent molar volume, Ṽ_1_, vs. α_*p*_ in silicone rubber MPF-50 (Machado et al., 1999).

Machado and co-workers (Machado et al., 1999) measured the permeability of pure liquid alcohols and hydrocarbons as a function of applied pressure in MPF-50, a commercial membrane made by cross-linked silicon rubber. They observed, especially for higher alcohols (i.e., isopropanol, butanol, and pentanol) and large hydrocarbons (n-pentane), a severe flux decline starting at 15 atm. For methanol such decline was less pronounced. To justify this behavior, Machado and co-workes invoked membrane compaction. However, in doing so, the authors recognized that flux decline at high pressures was significant especially for bulky solvents, i.e., solvent endowed with large molecular size. We believe their conclusions are somehow misleading, since membrane compaction is a purely mechanical effect that should take place regardless of penetrant size. Machado used the following empirical expression to fit flux vs. pressure curves:

(13) $$n_{1}=n_{1}^{0}\exp\left(-\alpha_{p}\Delta p\right)$$

where $n_{1}^{0}\;$is a pre-exponential factor and α_*p*_ is an empirical parameter that measures membrane compaction: the higher the membrane compaction, the higher α_*p*_. So, flux decline becomes faster with increasing α_*p*_ (that is, flux decline starts at lower pressures with increasing α_*p*_). Paul's theory can be used to rationalize this behavior, without invoking membrane compaction. Indeed, Equation 12 states that (i) at low Δ*p* flux is linear, as it can be demonstrated by replacing the exponential term with a first order Taylor expansion (i.e., $1-exp\left(-\frac{{Ṽ}_{1}\Delta p}{RT}\right)≅\frac{{Ṽ}_{1}\Delta p}{RT}$ when Δ*p* is small), and (ii) at sufficient high Δ*p*, the downward curvature of the flux-pressure curve becomes more pronounced as the solvent molar volume increases. Figure 6A shows the dimensionless flux, $n_{1}^{dim}$ , (i.e., $n_{1}^{dim}=\left[1-exp\left(-\frac{{Ṽ}_{1}\Delta p}{RT}\right)\right]$) as a function of Δ*p*, for different values of Ṽ_1_. The downward curvature of the flux-pressure curves becomes more pronounced with increasing permeant molecular size, as observed by Machado.

Interestingly, for any given couple of solvents, *i* and *j*, the $\alpha_{p}^{i}/\alpha_{p}^{j}$ ratio matches very well with the molar volumes ratio, Ṽ^*i*^/Ṽ^*j*^ (cf. Figure 6B). This analysis points out that the parameter α_*p*_ in the empirical Machado's model does not account for membrane compaction, as the authors state. According to Paul's theory, α_*p*_ is a measure of penetrant molecular size. So, occurrence of flux decline is not sign of membrane compaction, as commonly believed in the literature. Flux decline simply indicates that the system is approaching a condition where the driving force for solvent transport is maximum. When this maximum is reached, the flux attains its ceiling value. Moreover, the magnitude of flux decline increases with increasing permeant molecular size. So, the empirical Machado's model, if interpreted correctly, coincides with the rigorous Paul's model.

## Current OSN materials

**Figure 7:**
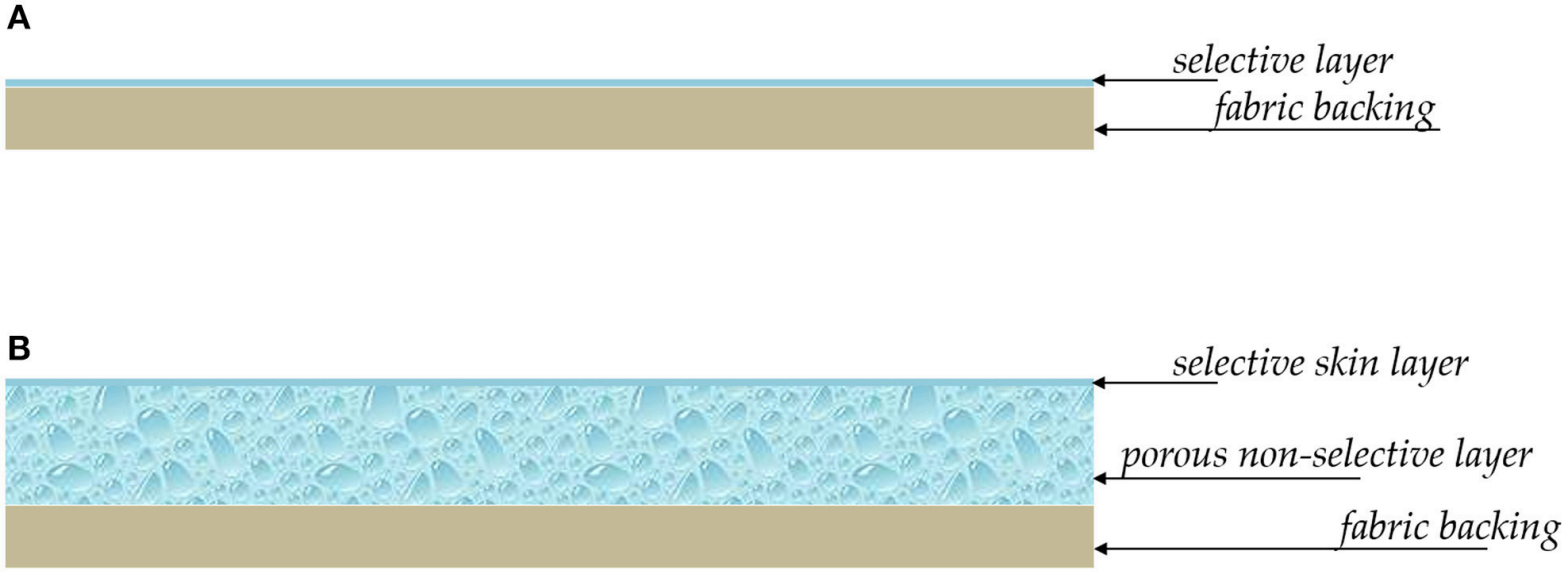
Structure of composite **(A)** and asymmetric **(B)** membranes for OSN.

OSN is a relatively young process, but with enormous potential for growth. Small scale applications of OSN are today in the pharmaceutical and cosmetic industry, as well as in refinery (dewaxing of lube oil) (Marchetti et al., 2014, 2017; Buonomenna and Bae, 2015). Composite membranes, consisting of a very thin (< 1 μm thick) active layer supported onto a porous backing, are often considered in the academic research and industrial practice (cf., Figure 7). The active layer and the backing support are made by different materials and have different structures and properties. They are combined together exploiting interfacial polymerization, as well as well-established spin coating or dip coating techniques. The support is generally made by polysulfone, polypropylene, polyimide or polybenzimidazole, depending on the environment the membrane has to face with. The active layer, which performs the separation, is generally made by cross-linked Lenzing P84 polyimide or cross-linked silicon rubber (PDMS) (Marchetti et al., 2014). The chemical structure of P84 polyimide and PDMS and their relevant chemical-physical properties are reported in Table 1. Alternatively, asymmetric membranes can be used (cf., Figure 7). Asymmetric membranes exhibit a dense skin, which performs the separation, supported onto a porous medium, made of the same material as the skin itself. They are prepared via phase-inversion, a process originally developed by Loeb and Sourirajan (Loeb and Sourirajan, 1963). A dilute solution of the membrane material in an appropriate solvent is cast onto a fabric backing. Then, the freshly cast membrane is soaked in a non-solvent bath where phase separation occurs. The polymer-rich phase generates the dense active layer, and the polymer-poor phase generates the porous support. Compared to composite membranes, asymmetric membranes exhibit lower resistance to physical aging and less stable properties during operation.

**Table 1:** Current standard materials for OSN: Lenzing P84 polyimide and PDMS.

	**P84 polyimide**	**PDMS**
	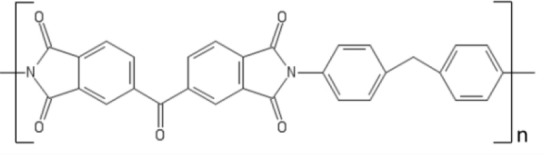	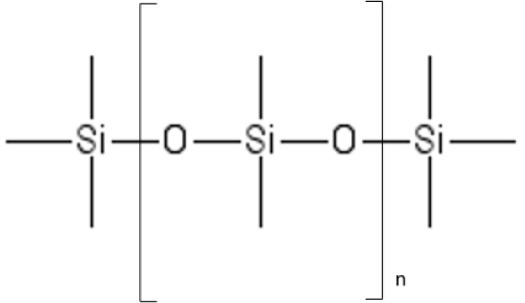
Glass transition temperature (°C)	329	−123
Young's modulus (GPa)	2.50	0.004
Methanol permeance^*^ [L/(h m^2^ bar)]	5.48	0.619
Source	(P84 product data, http://www.hppolymer.com/pdfs/Dimond%20wheel%20brochure.pdf; Darvishmanesh et al., 2010)	Lotters et al., 1997; Postel et al., 2013

* *At room temperature*.

Standard membrane materials for OSN are polyimides (White, 2002) and cross-linked silicone rubber (Postel et al., 2013), even though new materials, such as polybenzimidazole and PIMs, are emerging as promising candidates (Valtcheva et al., 2014, 2015). Unlike gas separation, OSN currently relies on a limited number of membrane materials, since polymers capable to withstand organic environments are rare.

Since the pioneering studies conducted by White in the early 2000's (White, 2001, 2002), Lenzing P84, a polyimide obtained by the condensation of 2,4-diidocyanato-1-methylbenzene and 1,1′-methylenebis(4-isocyanatobenzene) with 5,5′-carbonylbis(1,3-isobenzofurandione), is currently the standard membrane material for OSN (Silva et al., 2005; See-Toh et al., 2007a, 2008). Lenzing P84 films can be fabricated via solution casting from DMF, NMP, DMSO and DMAc solutions. Due to its fully aromatic, rigid structure, Lenzing P84 exhibits long lasting resistance to several solvents, such as hydrocarbons, toluene, alcohols and ketones. White prepared asymmetric membranes based on Lenzing P84 to separate linear from branched alkanes in toluene solutions. He showed that pristane, a saturated hydrocarbon bearing 19 carbon atoms, exhibited lower flux through Lenzing P84 relative to n-docosane, a linear saturated hydrocarbon bearing 22 carbon atom. This behavior is interesting, since pristane has lower molecular weight and molar volume relative to docosane. White attributed this behavior to the larger cross-section area of pristane, which increases the frictional resistance offered by the polymer to molecular diffusion. Based on this picture, pristane is better rejected than docosane due to its slower diffusion through the membrane material (White, 2002).

Afterwards, White used Lenzing P84 to separate aromatic from aliphatic aromas: interestingly, aromatic compounds exhibited higher fluxed than aliphatic ones. This behavior was attributed to the sorption contribution. Indeed, aromatic compounds interact more favorably with the aromatic polymer backbone than aliphatic species do. Such favorable thermodynamic interaction enhances the solubility of aromatic over aliphatic species in the membrane, which, based on the solution-diffusion model, justifies the experimental findings (White, 2002).

To improve the stability in aggressive aprotic solvents (e.g., THF, DMF and NMP), in which polyimides are soluble, Livingston and co-workers crosslinked Lenzing P84 using aliphatic diamines (ethylenediamine, propanediamine, hexanediamine, and octanediamine) in methanol solution. The cross-linking mechanism was described in detail by Liu et al. (2001). Specifically, the amino groups available on the cross-linker react with the imide groups on the polymer backbone to form amide moieties. The matrix swelling induced by methanol sorption is an essential pre-requisite for the formation of cross-linking. Indeed, if the polymer is not swollen enough, the reaction between the amino and the imide groups can be very slow. The method devised by Livingston produces uniform cross-linking throughout the whole membrane thickness. Other methods based on the radical-initiated cross-linking cannot be exploited, since they would effectively cross-link just the membrane surface, thus causing the membrane failure in aprotic acids. Cross-linked Lenzing P84 exhibited excellent stability in tetrahydrofuran (THF), N-methyl pyrrolidone (NMP), and dimethylformamide (DMF). FTIR analysis demonstrated that no morphological changes occurred in the membrane after soaking in these solvents for 120 h (See-Toh et al., 2007b). Obviously, the gain in stability upon cross-linking is accompanied by a decrease in permeability, caused by the enhanced chain packing.

Cross-linked PDMS has been tested to concentrate dyes from organic solutions and remove solvents from vegetable oils (Subramanian et al., 2001, 2003; Koike et al., 2002; de Morais Coutinho et al., 2009). The main drawback for using PDMS is its poor resistance to aggressive solvents. Indeed, due to the high chain flexibility and weak rigidity, PDMS sorbs a significant amount of solvent (Favre et al., 1994; Whu et al., 2000; Sheth et al., 2003; Vankelecom et al., 2004), which prejudices the membrane stability.

Both Lenzing polyimide and PDMS suffer of several limitations. Lenzing PI exhibits, indeed, modest fluxes but high resistance to aggressive environments. In contrast, PDMS exhibits high fluxes but poor chemical resistance. So, new materials are sought to overcome such limitations.

In recent years, polybenzimidazole (PBI), commercially available under the trade name of Celazole®, emerged as a promising material for OSN. PBI, first synthesized by Vogel and Marvel (Vogel and Marvel, 1961), is prepared from isophthalic acid and 3-3′-diaminobenzidine. It exhibits a rigid, aromatic structure with frustrated chain mobility. Moreover, intermolecular hydrogen bonding acts as a cross-link and contributes to mitigate swelling and plasticization in the presence of organic liquids. PBI exhibits also unusual thermal stability, with a negligible mass loss (< 5%) upon exposure to 570°C. Such unique combination of rigidity, high glass transition temperature and unprecedented chemical and thermal resistance, makes PBI suitable for separations in chemically challenging environments (Borjigin et al., 2015). However, the lack of solubility in most of organic solvents makes it difficult manufacturing thin membranes based on PBI. This polymer is only soluble in aggressive solvents, such as dimethylacetamide (DMAc). To further improve solubility in DMAc, LiCl is added to the dope solution, which complicates the post-processing treatment of the membranes as, after fabrication, LiCl has to be removed. These issues hamper the use of PBI in the industrial practice.

**Figure 8:**
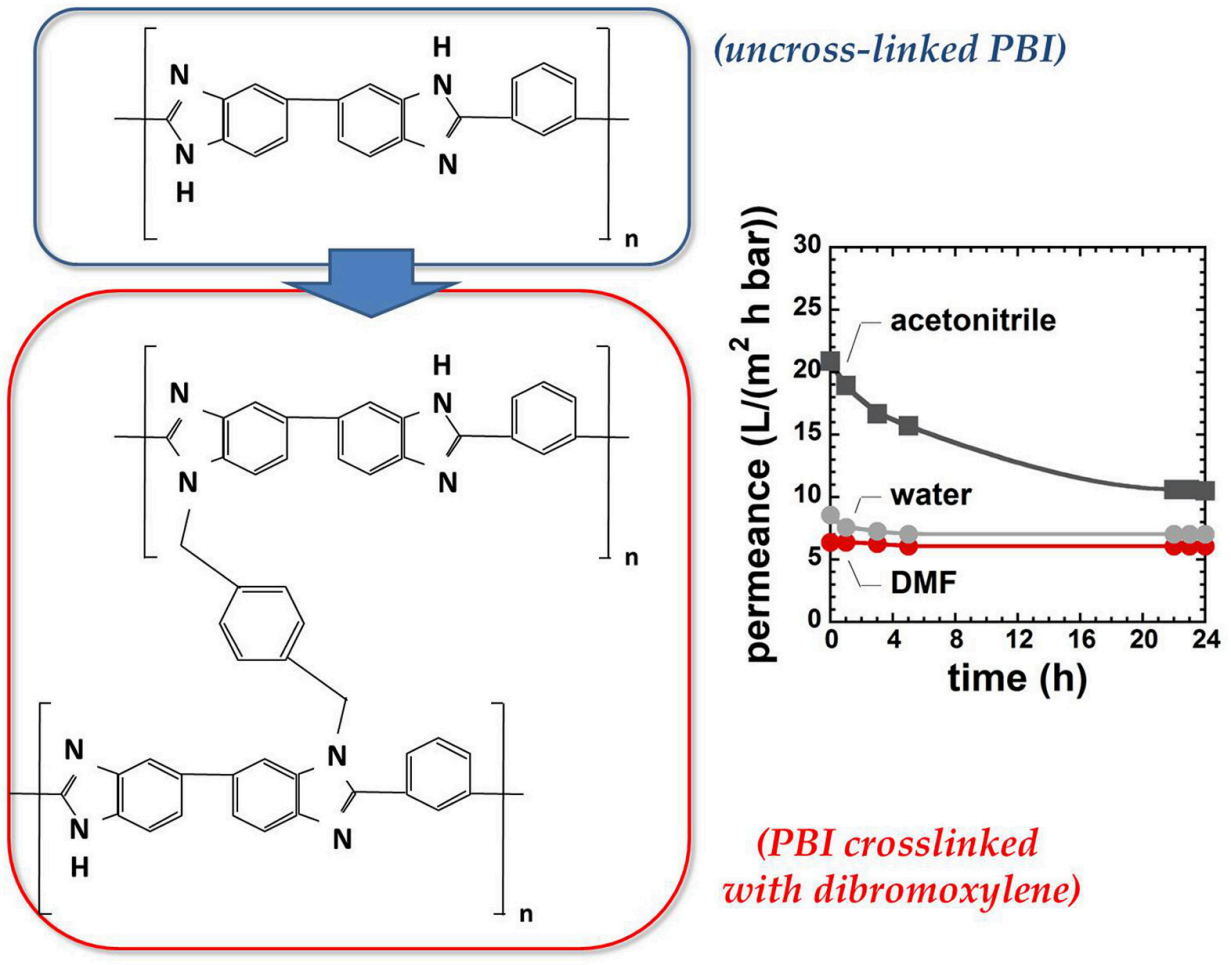
Structure and properties of novel PBI membranes cross-linked with dibromoxylene. Adapted from Valtcheva et al. (2014), with permission of *Elsevier*.

Livingston and co-workers developed cross-linked asymmetric membranes based on PBI with superior resistance in acid environments (Valtcheva et al., 2014). PBI was cast from a DMAc solution onto a non-woven polypropylene support. The nascent membrane was then soaked in water to generate phase separation and induce the asymmetric structure. Following this protocol, membranes were cross-linked upon immersion in a hot solution of dibromobenzene in acetonitrile (cf., Figure 8). Interestingly, the resulting cross-linked membranes exhibited good separation performance for several PEG markers in acetonitrile and dimethylformamide, as well as excellent dimensional stability even in hydrochloric acid solutions.

Since the pioneering work by Sourirajan in the early 60s, cellulose acetate and its derivatives have been, for at least two decades, the most studied materials for OSN applications. However, due to its poor resistance to aggressive solvents, cellulose acetate could only be used to separate a limited number of mixtures, such as water/alcohol. Moreover, due to the presence of crystalline domains, solvent flux through cellulose acetate membranes is relatively low. Recently, Vanherck et al. developed a new strategy to enhance the permeability of cellulose acetate OSN membranes without sacrificing the selectivity (Vanherck et al., 2011). They exploited the principle of photothermal heating by embedding gold nanoparticles (GNPs) into a cellulose acetate membrane. In this way it is possible to increase the local temperature in the membrane during operation by light irradiation, which, in turn, significantly enhances solvent flux. Such increase in solvent transport rate is essentially ascribable to an increase in diffusion coefficient. Indeed, solvent mobility coefficient through the membrane increases exponentially with increasing temperatures, due to the reduction of frictional resistance offered by polymer chains to penetrant diffusion. In contrast, the effect of temperature on the sorption coefficient is much weaker. The same principle was exploited by Nakai to improve by 37% CO_2_ permeability through cellulose acetate (Nakai et al., 2002).

The method proposed by Vanherck leverages on the gold nanoparticles ability to absorb light and convert it into heat (Daniel and Astruc, 2004). Such an effect becomes especially efficient at specific wavelengths, where surface plasmon resonance occurs. Golden nanoparticles were generated directly into a pre-made cellulose acetate membrane using the protocol reported by Huang (Huang et al., 2005). The amount of nanoparticles in the membrane was varied between 0 and 2% wt. To run nanofiltration experiments, Vanherck modified a dead-end filtration cell by building a glass window on top of it, to allow an argon-ion laser beam (wavelength = 514 nm) to irradiate the active membrane area (Vanherck et al., 2011).

Interestingly, water and alcohol permeability increased up to 15 and 400%, respectively, upon laser irradiation, with no detectable effects on solute (bromothymol blue) rejection. Moreover, solvent permeability returned to its original value after the laser was turned off, indicating that the observed change in transport properties was only due to the reversible membrane heating and not to permanent structural modifications. The much larger permeability improvement observed for alcohols was attributed to the lower specific heat of alcohols relative to water, which permits a more rapid heating of the swollen membrane. Likewise, alcohols exhibit lower thermal conductivity relative to water, so, while flowing through the membrane, they remove less heat.

## Molecular factors affecting solute and solvent transport in OSN membranes

The vast majority of research efforts on OSN focused, so far, on applications: commercially available or newly synthesized materials were used to measure solvent flux and solute rejection and such measurements helped to assess the suitability of those materials for specific applications. However, little information is available about molecular factors affecting solute and solvent transport in OSN membranes. The transport mechanism itself is object of discussion, as some researchers assume a solution-diffusion mechanism, others a pore-flow mechanism (Marchetti and Livingston, 2015; Ben Soltane et al., 2016). However, many factors, above all the existence of a permeability-selectivity trade-off, suggest that OSN membranes work based on the solution-diffusion mechanism. This conclusion was confirmed unequivocally by a series of simple but effective experiments performed by Paul in the early 70s. Specifically, Paul measured solvent permeability through a stack of several membranes. After reaching steady-state, the membranes were rapidly removed from the permeation cell and weighed. The experimental solvent concentration profile in the stack was in excellent agreement with that predicted assuming a solution-diffusion transport mechanism (cf. Figure 5, Paul and Ebra-Lima, 1971).

Fundamental structure-property correlations are available for gas separation membranes, which makes it possible to design polymers with pre-assigned transport properties. For example, use of polyethers for CO_2_ separation leverages on the high thermodynamic affinity between CO_2_ molecules and polar ether groups, which enhances CO_2_ sorption and, according to Equation 8, CO_2_ selective permeability (Bondar et al., 1999). So, membranes for gas separation are engineered by properly tuning solubility and diffusivity of the target penetrant in the membrane material. This goal is achieved, in turn, by tuning the polymer chemistry, that is, using monomers bearing specific functional groups.

The lack of information about the role of sorption and diffusion coefficients on solvent and solute transport is actually a roadblock toward the rational design of smart OSN membranes. Nevertheless, in this section we identify, based on the few data available, the main molecular factors that influence solute and solvent transport in OSN membranes.

From a theoretical standpoint, small molecule sorption in polymers is a phase equilibrium problem (Galizia et al., 2012). Sorption equilibrium is reached when penetrant chemical potential in the external fluid phase equates that in the polymer mixture. As chemical potential contains, by its definition, an enthalpic (i.e., energetic) and an entropic contribution, small molecules sorption in polymers is influenced by these two effects (Galizia et al., 2012). Enthalpic effects are related to polymer-penetrant interactions and, as a rule of thumb, favorable interactions translate in high penetrant solubility in the membrane material. Entropic effects are related to penetrant size. As general rule, sorption decreases with increasing penetrant size. Indeed, absent specific interactions, it is less and less likely to accommodate bulkier penetrant molecules in the polymer matrix than smaller penetrant molecules.

Vankelecom et al. (1997) and Cocchi et al. (2015a) reported sorption and diffusion coefficients of several liquids in rubbery PDMS. Alkanes solubility in PDMS decreases linearly with the number of carbon atoms, indicating that sorption is affected essentially by entropic factors. In contrast, solubility of polar alcohols in PDMS follows a non-monotonous trend with the number of carbon atoms, as it increases moving from ethanol to 1-butanol, and then decreases linearly going from 1-butanol to higher alcohols, following the same trend observed for alkanes. This phenomenon was justified based on the interplay between enthalpic and entropic effects. Indeed, PDMS exhibits poor thermodynamic affinity with lower polar alcohols. The strong repulsive interactions between –OH polar groups and the non-polar PDMS backbone strongly oppose to alcohol dissolution in the polymer. When the length of the alkyl tail increases, the affinity between alcohols and PDMS increases, which justifies the increase in solubility observed going from methanol to 1-butanol. When the alkyl tail is long enough, the polar –OH group is sterically shielded, so alcohols start to behave as alkanes and their solubility in PDMS decreases with increasing penetrant size. So, the increase in solubility from methanol to 1-butanol is driven by enthalpic (i.e., energetic) effects, and the subsequent decrease is driven by entropic effects.

Vankelecom demonstrated that the solubility of organic liquids in PDMS decreases linearly with increasing the difference between the penetrant and polymer Hildebrandt solubility parameter, δ−δ_*PDMS*_ (Vankelecom et al., 1997). Afterwards, Cocchi showed that a better linear correlation is obtained if penetrant solubility in PDMS is plotted against $M_{w}^{0.75}{\left(\delta -\delta_{PDMS}\right)}^{2}$, where *M*_*w*_ is the penetrant molecular weight (Cocchi et al., 2015a). Indeed, while the correlation reported by Vankelecom accounts just for energetic factors, that reported by Cocchi accounts for both energetic (through δ) and entropic (through *M*_*w*_) factors. Doing so, sorption data for hydrocarbons, alcohols and several other organic liquids fall on a master curve. Unfortunately, such analysis is fairly absent in the literature for glassy polymers. Cocchi et al. (2015b) also reported mixed solvent-solute solubility data in PDMS. In mixed conditions, solute solubility in the membrane is greatly enhanced, up to 25 times, compared to pure solute solubility, which indicates that real solvent-to-solute solubility-selectivity is lower than that predicted on the basis of pure component sorption data. The large enhancement in solute sorption in mixed conditions was justified based of the high degree of membrane swelling induced by the solvent.

Diffusivity-selectivity data for solute-solvent mixtures are virtually absent in the literature. Small molecules diffusion coefficients in polymers generally decrease with increasing penetrant size (Galizia et al., 2017b). The critical volume, as well as the kinetic diameter or squared kinetic diameter have been used as a measure of penetrant size. Interestingly, diffusion coefficients of liquid penetrants in PDMS do not always correlate to penetrant size. For example, as reported by Cocchi et al. (2015a), acetone diffusivity in PDMS is larger than water diffusivity, despite water (kinetic diameter = 0.265 nm) is a smaller molecule than acetone (kinetic diameter = 0.50 nm). This behavior was explained considering that acetone is sorbed to a greater extent than water by PDMS, so acetone diffusion occurs in a much more swollen membrane, which offers a lower frictional resistance to penetrant transport. However, subtle interactional effects influence penetrant diffusion in polymers. As discussed above, penetrants diffusion coefficient in polymers is the product of a purely kinetic quantity, i.e., the mobility coefficient, *L*_1_, which quantifies the frictional resistance offered by the polymer matrix to penetrant diffusion, and a thermodynamic factor, related to polymer-penetrant interactions. The thermodynamic factor is higher than one when polymer-penetrant interactions are thermodynamically favorable, and less than one when such interactions are thermodynamically unfavorable. Due to the strong hydrophobicity of PDMS, acetone-PDMS affinity is much higher than water-PDMS affinity, so the thermodynamic contribution to acetone diffusion in PDMS is expected to largely exceed that of water (Favre et al., 1994; Singh et al., 1998), which would justify the experimental findings reported by Cocchi.

## Recent advances and future directions in OSN

### Polybenzimidazoles

In this section, we identify new generation materials for OSN and outline applications that will likely drive OSN research in the next decades. As mentioned in section Current OSN Materials, polybenzimidazole exhibits unprecedented thermal, mechanical and chemical stability, however, some drawbacks limit its use in the industrial practice. Above all, the poor chain mobility caused by the rigid diaminobenzidine segments, combined with the efficient chain packing produced by intermolecular hydrogen bonds, reduces small molecule permeability in PBI. As a matter of fact, PBI exhibits quite low fluxes (i.e., low productivity), which makes it unappealing for practical applications. To circumvent these issues, Riffle and coworkers synthesized a substituted PBI with improved processability and higher permeability relative to commercial PBI (Borjigin et al., 2015). They replaced the linear, rigid diaminobenzidine monomer with 3,3′, 4,4′ tetraaminodiphenylsulfone (TADPS). Such monomer exhibits higher flexibility relative to diaminobenzidine, due to the presence of the sulfonyl linkage between diaminophenyl groups (cf. Table 2). Moreover, the kinked structure of TADPS-PBI induced by sulfonyl groups frustrates chain packing and enhances small molecules sorption and transport. Interestingly, substituted PBI exhibits improved solubility in solvents (DMAc, DMSO, NMP), while maintaining chemical, mechanical and thermal stability comparable to those exhibited by commercial PBI. H_2_ and CO_2_ permeability coefficients in TADPS-PBI and in commercial PBI were fairly similar, but TADPS-PBI exhibited higher H_2_/CO_2_ selectivity. This fact is significant, since the improved flexibility induced by sulfonyl groups did not sacrifice the membrane performance. Investigation of substituted PBI for OSN application is underway.

**Table 2:** New materials for OSN application.

**Material**	**Structure**
PBI	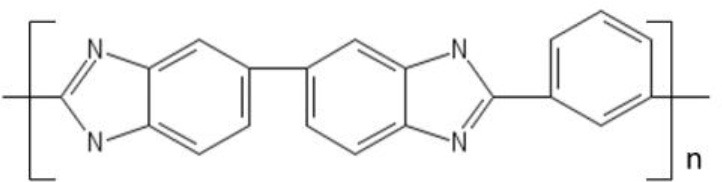
TADPS-PBI	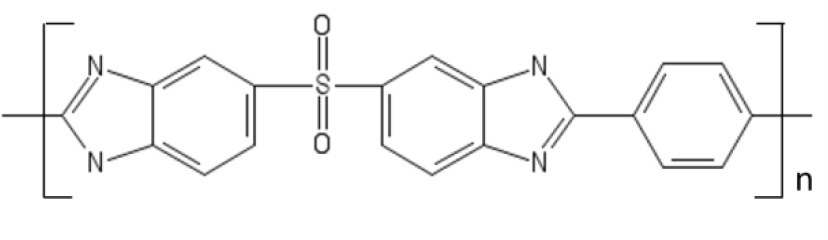
PIM-1	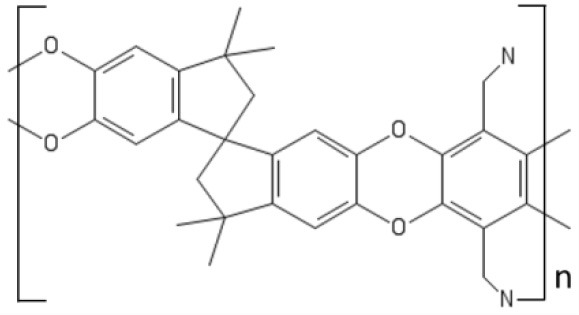
PIM-8	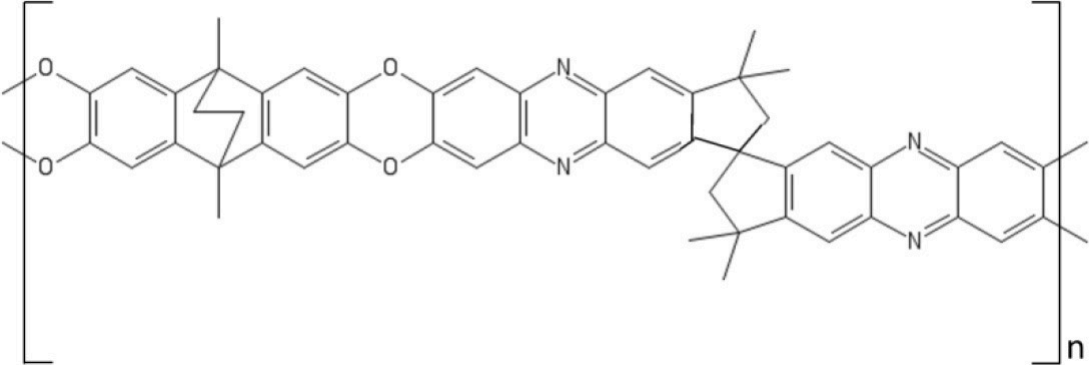
PTMSP	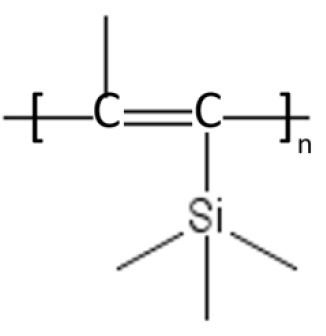
Teflon® AF	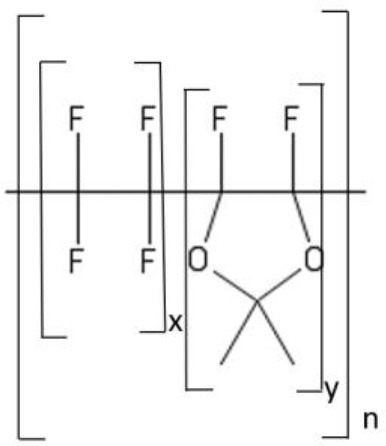
Pebax	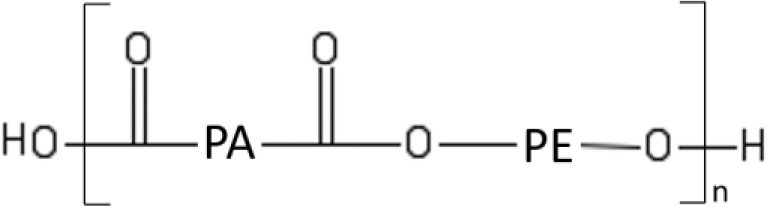
Polyarylates	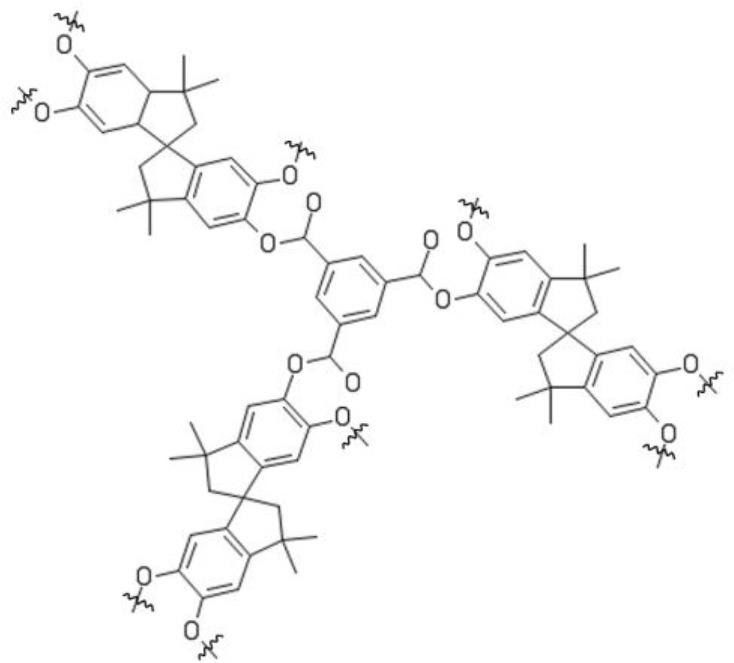

### Polymers of intrinsic microporosity (PIMs)

The most known member of this family of polymers, PIM-1, first synthesized by Budd and McKeown in 2004, is prepared by the nucleophilic substitution reaction of tetrafluoro-phathalonitrile with 5,5,6,6-tetrahydroxy-3,3,3,3-tetramethyl-1,1′-spirobisindane. The spiro-center of 5,5,6,6-tetrahydroxy-3,3,3,3-tetramethyl-1,1′-spirobisindane is responsible for the contorted structure exhibited by this polymer. PIM-1 exhibits a glass transition temperature above 450°C and a three-dimensional ladder structure, which frustrates chain packing and produces interconnected free volume cavities whose dimension is < 2 nm. Consistently, the BET surface area is very high, up to 800 m^2^/g. Due to this ladder-type, rigid structure, PIMs are poorly soluble in solvents, except THF and chloroform (Budd et al., 2004).

A drawback for using PIMs in OSN is their tendency to swell in the presence of solvents (Ogieglo et al., 2017), such as acetone and toluene, which produces very high flux but poor solute rejection. Several attempts have been made to synthesize alternative PIMs, but only a limited number of monomers is able to give polymers with a sufficiently high molecular weight (Sanders et al., 2013). Combining PIM-1 with other polymers could be, in principle, another route to improve its properties, but serious compatibility issues were reported. Gao et al. coated a PVDF support with a thin PIM-1 layer, but the adhesion of the active layer to the substrate was poor (Gao et al., 2017).

Currently, research efforts are underway to improve PIMs resistance to solvents by cross-linking or chemical modification. Fritsch (Fritsch et al., 2012) blended PIM-1 with polyethyleneimine (PEIm) in THF solution. Then, poly(ethylene glycol diglycidyl ether) (PEGDEG) was added to the solution. PEGDEG cross-linked the blend by reacting with the PEIm amino groups. Cross-linked PIM-1-PEIm membranes exhibited much better dimensional stability in solvents. Moreover, blending with rubbery PEIm reduced significantly the PIM-1 brittleness.

Livingston and coworkers developed composite membranes made of a 140 nm thick PIM-1 layer supported on PAN or alumina, which exhibited excellent resistance to physical aging after annealing for several hours at 150°C (Gorgojo et al., 2014). Remarkably, solvent permeability was almost 2 orders of magnitude higher than in commercial membranes based on Lenzing P84 polyimide. Unexpectedly, solvent permeance decreased with decreasing the active layer thickness below 140 nm. Indeed, as demonstrated by light interferometry, chain packing is enhanced in ultrathin films.

Very recently, PIMs emerged as promising materials for isomer separation. The latter is among the most challenging separations, as isomers exhibit like size and very similar chemical-physical properties, which makes it difficult developing membranes with adequate levels of selectivity. Such separations are highly demanded in the pharmaceutical industry, where isomers of a given compound often exhibit different therapeutic properties. Intrinsically chiral materials exhibited high selectivity for chiral separations. Weng and coworkers (Weng et al., 2015) synthesized intrinsically chiral PIMs with spectacular selectivity for several isomers of practical deal. Chiral ladder (+)PIM-CN was prepared by condensation reaction between enantiomerically pure 5,5′,6,6′-tetrahydroxy-3,3,3′,3′-tetramethyl-1,1′-spirobisindane and 2,3,5,6-tetrafluorophthalonitrile in DMF, in the presence of K_2_CO_3_. Selectivities as high as 14 were observed for some chiral separations (e.g., the resolution of R,S mandelic acid and R,S-binol). Interestingly, sorption experiments revealed that solubility-selectivity is equal to one, so the selective permeation of isomers through this material is solely driven by the diffusion contribution. However, the fundamental origin of this behavior is still unknown.

Future research efforts will likely focus on PIMs different than PIM-1. For example, PIM-8 was synthesized by Ghanem et al. by polymerization of 2,3,6,7-tetrahydroxy-9,10-dimethyl-9,10-ethanoanthracene (Ghanem et al., 2008). PIM-8 exhibited a 50% higher selectivity for linear vs. branched alkanes relative to PIM-1. Cook and coworker attributed this behavior to a narrower and much regular size distribution of free volume cavities in PIM-8 (Cook et al., 2018).

### Perfluorinated glassy polymers

Perfluorinated polymers are soluble in a limited number of fluorinated liquids and exhibit excellent resistance to the vast majority of organic solvents. Recently, Chau reported solvent permeability and solute rejection data for a series of perfluorodioxole copolymers, such as Hyflon®AD and Cytop® (Chau et al., 2018). Solvent (methanol, hydrocarbons, aromatics, THF) sorption in perfluorodioxole copolymers is about 1% wt, which is much lower than in other glassy polymers. Interestingly, n-heptane sorption in perfluorodioxole copolymers is much larger, 2.5% wt, which enhances n-heptane flux far above that of other organic solvents and reduces substantially solute rejection. The molecular origin of this behavior was tentatively attributed to the higher matrix dilation induced by bulky n-heptane molecules. This explanation, however, appears not convincing on the basis of the relatively low n-heptane sorption level. This example demonstrates the urgent need of fundamental transport studies in glassy OSN membranes.

### Block copolymers with hard and soft segments

Poly(ether block amide), commercially available under the trade name Pebax®, is a thermoplastic block copolymer made by rigid polyamide segments, which provide mechanical rigidity, interspaced with highly permeable, rubbery polyether segments. Pebax® has shown superior performance in gas separation (Bondar et al., 2000), and recently it raised attention in OSN (Aburabie and Peinemann, 2017). The relative amount of glassy and rubbery segments can be tuned, resulting in different Pebax® grades exhibiting different transport and mechanical properties (Bondar et al., 1999, 2000). The role of the glassy segments consists in providing mechanical strength, reducing the swelling of the highly permeable rubbery blocks and maintaining good levels of dimensional stability and selectivity. Pebax® can be cross-linked upon immersion in toluene diisocyanate (TDI) solutions, which promotes the formation of urethane linkages between the hydroxyl groups on the polyether segments and the carbonyl groups of TDI (Aburabie and Peinemann, 2017). The cross-linking mechanism proposed by Aburabie was confirmed via DSC analysis. Compared to the pristine, uncross-linked sample, the endothermic peak attributed to the fusion of polyether blocks in the cross-linked sample shifted from 50 to 80°C, whereas the peak assigned to the polyamide blocks did not show any change. This result confirms that the cross-linking is produced by the reaction of TDI with the polyether blocks, as explained above (Aburabie and Peinemann, 2017).

Aburabie showed that ethanol permeance of composite membranes fabricated by coating a PAN support with Pebax® was 8 times reduced relative to the PAN support alone (Aburabie and Peinemann, 2017). Moreover, the Pebax® active layer was not able to reject brilliant blue in ethanol solution, due to the excessive membrane swelling. A significant enhancement in separation performance was observed after cross-linking. The best results were obtained using a 2% wt cross-linker concentration and a reaction time of 30 min. In this condition, rejection of brilliant blue was about 95% and ethanol permeance was 0.1 L/(m^2^ h bar). When the reaction time was larger than 30 min, the membrane assumed a strongly packed structure, likely due to a very high degree of cross-linking. In this condition, ethanol flux was fairly zero even at pressures of 20 bar.

Interestingly, Pebax®/PAN membranes cross-linked with 2% TDI for 30 min exhibit 100% rejection of olive oil from a 10% wt olive oil solution in acetone (Aburabie and Peinemann, 2017). Thus, Pebax® appears suitable for application in the food industry, where organic solvents are often used to extract vegetable oils from seeds. Currently, solvent recovery from edible oils is performed by distillation, which can alter the organoleptic properties of oils.

The main drawback for using Pebax® in OSN is its tendency to swell in water and alcohols, which leads to lower selectivities. Water flux through uncross-linked Pebax® membranes increases by almost 50% after 20 h of soaking in water. In contrast, membranes cross-linked with TDI do not exhibit any increase in water flux after swelling in water for 20 h (Aburabie and Peinemann, 2017). So, cross-linking with TDI appears a viable route to produce stable Pebax® membranes for OSN applications.

### High free volume polyacetylenes

Substituted polyacetylenes, such as poly(trimethylsilyl propyne) (PTMSP) and poly(methylpentyne) (PMP), exhibit a rigid, glassy structure and bulky functional groups that produce inefficient chain packing, with fractional free volume levels up to 30% (Tanaka et al., 1985; Merkel et al., 2000; Galizia et al., 2014). Composite PTMSP membranes exhibited ethanol permeances as high as 17 L/(m^2^ h bar), which is one of the highest values reported in the literature so far (Grekhov et al., 2012; Volkov et al., 2013). However, freshly cast PTMSP membranes exhibit an accelerated physical aging even when they are fabricated as bulky films (Nagai et al., 2000). This behavior is due to the strong departure of this material from equilibrium conditions, as witnessed by the high free volume level. Recently, Lau and co-workers discovered that physical aging of PTMSP and PMP can be stopped upon addition of microporous PAFs (Porous Aromatic Frameworks) (Lau et al., 2014). Specifically, microporous PAFs exert a chain threading effect by partially sorbing the polymer in their pores, which freezes the polymer structure and keeps the polymer chains in their original position. Such beneficial effect completely stops compaction and densification of high free volume polyacetylenes. This result represents a breakthrough and could bring the scientific community to reconsider application of polyacetylenes in OSN. In principle, to further improve its stability in chemically aggressive environments and delay physical aging, PTMSP can be cross-linked with 3,3′-diazidodiphenylsulfone. However, as noted by Kelman et al., chemical cross-linking reduces gas permeability by 70% relative to uncross-linked PTMSP, which makes this material no longer attractive for industrial applications (Kelman et al., 2007). As a matter of fact, no systematic study about the use of cross-linked PTMSP in OSN is available in the literature.

### Isoporous membranes

Developing isoporous membranes for OSN, i.e., membranes with a very uniform size and distribution of free volume cavities (improperly defined “pores”), will be an important goal over the next years (Yu et al., 2014). While this approach is under investigation for gas separation membranes (Abetz, 2014), a few authors attempted to exploit it for OSN. The actual challenge is that free volume architecture can be disrupted upon exposure to aggressive solvents, thus thwarting the efforts made to synthesize materials with specifically-sized free volume cavities.

**Figure 9:**
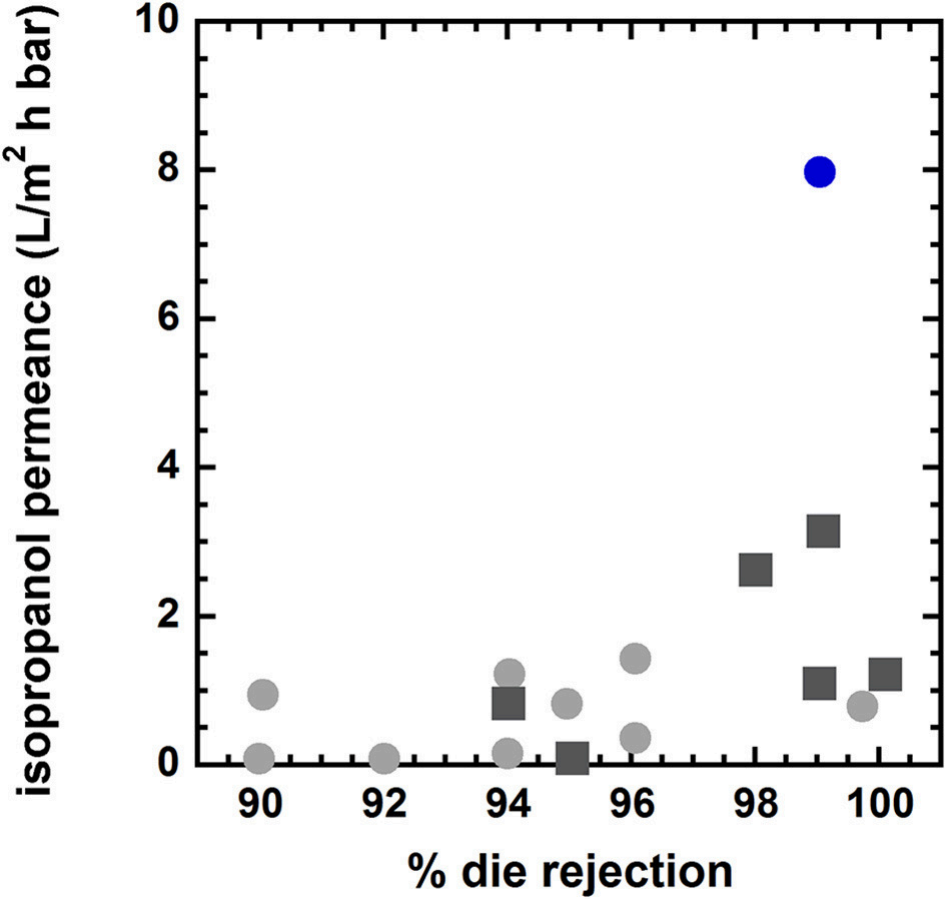
Isopropanol permeance vs. rose Bengal rejection at room temperature and Δp = 30 bar. Solid gray circles: conventional asymmetric membranes. Solid black squares: conventional composite membranes. Solid blue circle: polyarylate synthesized using non-planar, contorted monomers (Jimenez-Salomon et al., 2016). Adapted from Jimenez-Salomon et al. (2016), with permission of *NatureResearch*.

Recently, Jimenez-Salomon exploited interfacial polymerization to fabricate highly permeable and selective polyarylate nanofilms, < 20 nm thick, onto cross-linked polyimide nanofiltration membranes (Jimenez-Salomon et al., 2016). They used contorted monomers, such as 5,5′,6,6′-tetrahydroxy-3,3,3′,3′-tetramethylspirobisindane (TTSBI) and 9,9-bis(4-hydroxyphenyl)fluorine (BHPS) to prepare polyarylates with controlled and specifically-sized free volume elements. During polymerization, TTSBI units acquire a non-coplanar orientation, which leads to polymers having three-dimensional structures and interconnected free volume elements. This picture was confirmed by molecular simulations. For comparison, they also synthesized polyarylates with planar, non-contorted monomers, such as dihydroxyanthraquinone and 1,3-benzenediol, which exhibited much lower permeance but similar selectivity relative to materials containing contorted monomers. Interestingly, isoporous polyarylates surpass abundantly the upper bound in several applications. As shown in Figure 9, isopropanol permeance exhibited by polyarylates is twice that of conventional asymmetric and composite membranes for OSN, and dye (rose Bengal) rejection is over 99%.

Another possible approach for the design of polymers with controlled free volume architecture relies on using monomers bearing triptycene moieties. Recently, this approach has been successfully used to design gas separation membranes (Luo et al., 2016, 2018; Weidman and Guo, 2017; Weidman et al., 2017) endowed with unprecedented levels of permeability and selectivity. Triptycenes are three-dimensional, paddlewheel-like structures formed by three aromatic rings arranged at 120° on three different planes. Once incorporated into the polymer backbone, trypticenes disrupt chain packing and introduce an ultra-fine microporosity which enables for superior size-sieving ability. Specifically, the microporosity is provided via the internal free volume associated to each triptycene moiety. Moreover, unlike ordinary glassy polymers, whose free volume is purely conformational, the internal free volume of triptycene units is configurational and, as such, it is not collapsible. According to this physical picture, iptycene-based polymers exhibit uncommon resistance to physical aging (Luo et al., 2016, 2018; Weidman and Guo, 2017; Weidman et al., 2017). Potential use of such materials in OSN is fairly unexplored. The advantages for using iptycene-based polymers in OSN could be twofold. First, the fine and homogeneous internal free volume provided by triptycenes is inaccessible to solute molecules, but it is potentially accessible to solvent molecules whose diameter is < 4Å, which should guarantee high solvent flux and high solute rejection. Moreover, tough glassy polymers, such as polybenzoxazoles modified introducing triptycene moieties, would also guarantee outstanding resistance in harsh environments, which is an essential condition for applications in OSN.

### Hybrid materials

The most revolutionary application of OSN in the future will likely be the separation of isomeric solutes from organic solutions. This application is relevant in the pharmaceutical and petrochemical industry. For example, separation of hexane isomers is crucial to enhance gasoline octane number. Mixed matrices formed by selective fillers embedded in polymer membranes could make this application a reality. For example, ZIF-77 has been identified as a good candidate for isomer separations (Dubbeldam et al., 2012; Krishna and van Baten, 2017), but, surprisingly, no publication dealing with mixed matrix membranes containing ZIF-77 is available in the literature.

Recently, a new MOF with hydrophobic quadrilateral channels was synthesized via a solvothermal process from [Fe_3_(μ_3_-O)(COO)_6_] and 2,2-bis(4-carboxyphenyl)-hexafluoropropane (6FDA) and tested for separation of hexane isomers (Lv et al., 2018). The successful separation of n-hexane from its branched isomers relies essentially on solubility-selectivity, as linear n-hexane can be selectively sorbed by the [Fe_3_(μ_3_-O)(COO)_6_]-6FDA MOF, while sorption of branched isomers is sterically hindered. The sorption selectivity exhibited by the [Fe_3_(μ_3_-O)(COO)_6_]-6FDA MOF is 20 times higher than that of other sorbents, such as ZIF-8, zeolite 5A and zeolite β (Luna-Triguero et al., 2017). Moreover, this smart MOF exhibits very stable sorption capacity and selectivity after several sorption-desorption cycles.

Significant research efforts are being devoted to the fabrication of hybrid materials that surpass the permeability-selectivity upper bound. For example, Wu and coworkers incorporated MXenes nanosheets in hydrophilic poly(ethyleneimine) (PEIm) and hydrophobic silicon rubber (PDMS) (Wu et al., 2016). MXenes, first reported by Naguib et al. in 2014, are 2-D inorganic structures consisting of a thin layer of transition metals (Naguib et al., 2014). The resulting membranes were mechanically robust and exhibited higher alcohol permeability and much higher alcohol selectivity relative to neat polymers, thus surpassing the permeability-selectivity upper bound. Specifically, MXenes nanosheets contain polar -OH groups which enhance alcohol sorption and, based on the solution-diffusion model, alcohol permeability (Wu et al., 2016). For example, isopropanol permeability in PDMS-MXenes membranes is 162% higher than in neat PDMS. Moreover, the MXenes nanosheets block solute (e.g., PEG oligomers whose molecular weight ranged from 200 to 1000 Da) transport through the membrane, by making their diffusional pathway more tortuous, which produces a significant increase in solute rejection.

**Figure 10:**
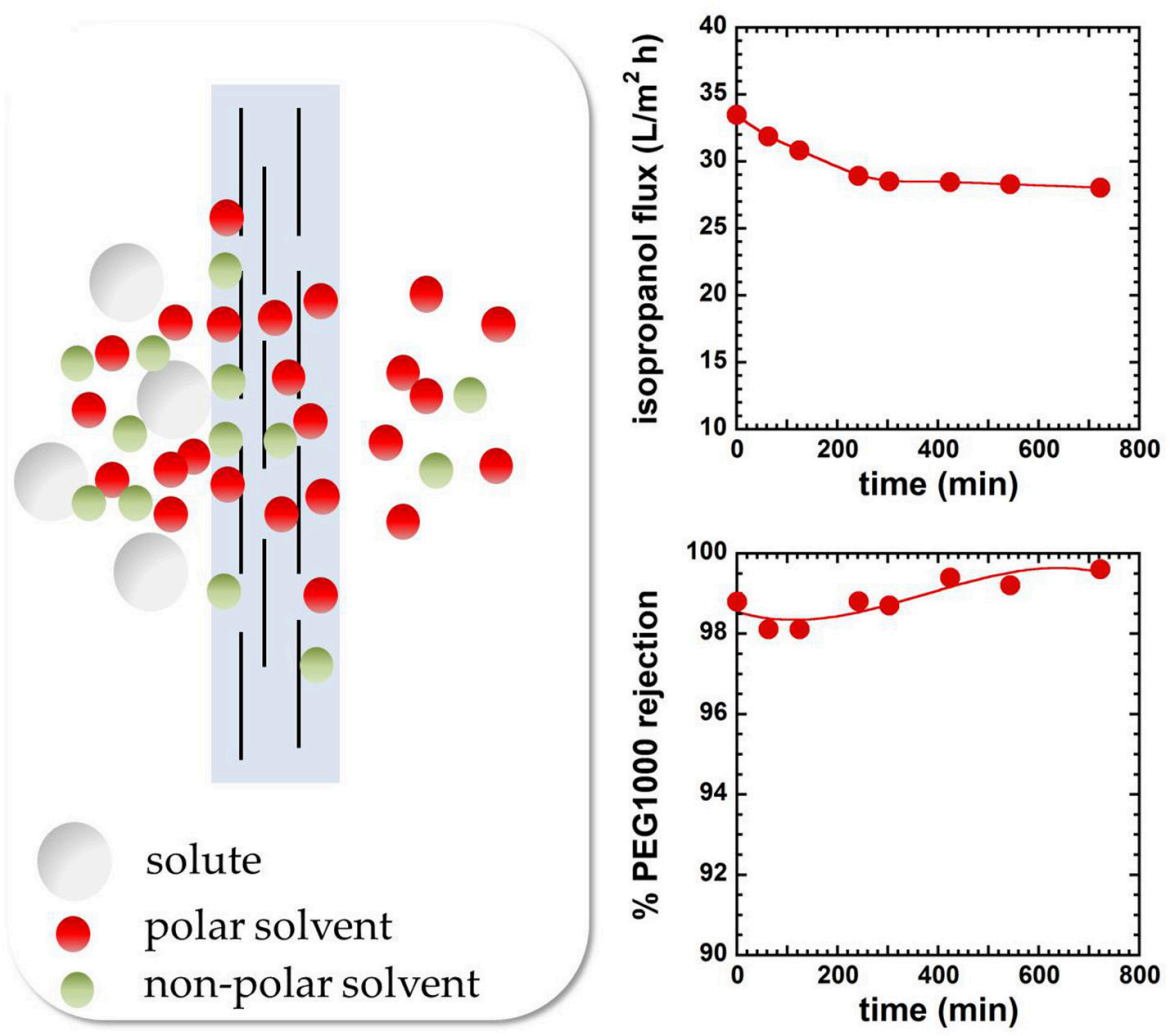
Structure and properties of mixed matrix membranes based on PEIm and MXenes (Wu et al., 2016). OSN experiments were run at room temperature and 10 bar using an isopropanol/PEG1000 solution. Adapted from Wu et al. (2016), with permission of *Elsevier*.

The isopropanol flux and PEG1000 rejection of the PEim-MXenes membrane at room temperature and 10 bar was tracked for 12 h (cf. Figure 10). Isopropanol flux declined by 20% (i.e., from 33.5 to 28 L/m^2^ h) in the first 4 h and then reached a pseudo-steady state value for the following 8 h. PEG1000 rejection changed from 98.8 to 99.2% within the same time frame. The flux decline was explained invoking membrane compaction and pore blockage by solvent molecules (Wu et al., 2016).

### Membranes based on preassembled nanoparticles

The phase separation process leads to membranes exhibiting a relatively broad distribution of free volume size. The possibility of tailoring the membrane microstructure so as to impart a well-defined free volume architecture has been explored is several ways. As discussed above, one possibility consists in using isoporous membranes. An alternative route to control the free volume architecture and create “manipulated” permeation pathways consists in preparing membranes based on preassembled nanoparticles. Several attempts have been made to deposit polymer nanoparticles on porous supports (Siddique et al., 2011). Nanoparticles can assemble in regular arrays, whose geometry influences the final transport properties of the membrane. Interstitial space between nanoparticles provides preferential permeation pathways. The “pore” size of these membranes can be controlled by tuning the nanoparticles size: if the nanoparticles size distribution is very uniform, the size of the interstitial channels is also uniformly distributed, which is an essential prerequisite to fabricate highly selective membranes (Siddique et al., 2011).

Siddique et al. deposited several layers of NIPAM (N-isopropylacrylamide)-HEMA(2-hydroxyethyl methacrylate) nanoparticles, whose diameter was 120 or 300 nm, onto a porous support via spin coating (Siddique et al., 2011). NIPAM-HEMA nanoparticles were first modified with acryloyl chloride to create polymerizable vinyl groups on their surface. The coating was then stabilized by UV cross-linking via radical polymerization. The support was prepared by casting a P84 polyimide solution onto a non-woven polyester backing. Phase separation of P84 was then induced upon immersion in a water bath. Following this step, the polyimide support was cross-linked upon soaking into a 1,6-hexanediamine-isopropanol solution. Finally, spin coating from methanol solutions was exploited to deposit a nanoparticle layer whose thickness ranged from 1 to 23 μm.

TEM micrographs show that moderate nanoparticle deformation takes place after deposition and UV cross-linking. Moreover, nanoparticles partially occupy the pores of the support membrane. Such observations are crucial to explain the results of nanofiltration experiments. Indeed, based on geometrical considerations, the theoretical pore size should be in the range 9–23 nm, so membranes coated with nanoparticles should not be able to separate molecules in the nanofiltration range (Siddique et al., 2011). However, rejection of styrene oligomers from toluene and acetone solutions was close to 100%, indicating that molecular separation in the nanofiltration range took place. The authors speculated that nanoparticle deformation is responsible for this behavior. As mentioned above, TEM analysis confirms this hypothesis. Interesting, solvent fluxes as high as 55 L/(m^2^ h) were measured, which led the authors to hypothesize that polystyrenes transport occurs in the interstitial spaces only, while solvent permeates through both the interstitial spaces and the nanoparticles (Siddique et al., 2011). Such hypothesis is consistent with the typical behavior exhibited by swollen NIPAM nanogels, which are highly permeable to small molecules, and practically impermeable to bulky solutes (Schild, 1992).

Interestingly, polystyrenes rejection increases with increasing the thickness of the nanoparticle layer, due to the narrower pore size distribution, and decreases with increasing nanoparticle diameter, due to the formation of larger interstitial spaces. So, the best membranes are obtained upon deposition of a relatively thick layer of small nanoparticles (Siddique et al., 2011). Deposition of nanoparticles exhibiting different sizes further improves solute rejection. Specifically, the mean pore size can be decreases by coating the support with larger nanoparticles first, and then with smaller nanoparticles.

### Nanopapers

Manufacturing of polymer nanofiltration membranes often requires the use of large amounts of toxic solvents and chemicals. Use of membranes made of cellulose or nanocellulose would provide a solution to the above mentioned issue, as these materials can be processed in aqueous solution (Mautner et al., 2014; Sukma and Culfaz-Emecen, 2018). Nanofibrillated cellulose in the paper form, also called nanopaper, exhibits outstanding mechanical properties and thermal stability, other than good barrier properties (Klemm et al., 2011; Mautner et al., 2014). Moreover, nanopapers exhibit nanopores whose size is comparable to that of a single molecule and, for this reason, they would be ideal candidates for OSN applications (Mautner et al., 2014). Recently, Mautner et al. prepared solvent stable OSN membranes from aqueous suspension of (2,2,6,6-tetramethylpiperidin-1-yl)oxy (TEMPO) oxidized nanofibrillated cellulose (Mautner et al., 2014). Addition of trivalent salts, such as AlCl_3_, to the aqueous suspension induces the flocculation of nanofibrils by changing their surface charge. Specifically, trivalent cations are sorbed on the negatively charged nanofibrils surface, causing a decrease of the ζ-potential (Mautner et al., 2014). Nanofibrils compaction leads to the formation of self-standing membranes that have shown organic solvents permeances up to 100 L/(h m^2^ MPa) and retention of polystyrene close to 100%.

Interestingly, organic liquids (n-hexane, tetrahydrofuran) permeance was larger than that of water, irrespective of the hydrophilic behavior of nanocellulose (Mautner et al., 2014). The molecular origin of this behavior is still unknown, since permeability coefficients were not deconvoluted into their elemental sorption and diffusion contributions. Moreover, water permeance decreased from 47 to 5 L/(h m^2^ MPa) after 1 h of operation (Mautner et al., 2014). The authors attributed this behavior to membrane compaction under pressure.

Interestingly, the average pore size is equal to the nanofibrils diameter, so the porosity of these membranes can be finely tuned by changing the size of the cellulose nanofibrils (Mautner et al., 2014).

## Conclusions

Organic solvent nanofiltration is a new paradigm in the chemical industry. Despite it is intrinsically safe, energy efficient and scalable, OSN is today one of the most poorly understood processes at a fundamental level. Factors that limit its exploitation in the industry are:

*i)* The lack of fundamental knowledge about solute and solvent transport mechanism. Moreover, the few elemental transport data available are often interpreted in a too simplistic way, which contributes to the spread of misleading conclusions in the literature.
*ii)* The lack of materials capable to tolerate chemically challenging environments.

While recent research efforts contributed to address the latter point, fundamental understanding of OSN remains poor and incomplete. For example, diffusion coefficients in solvent swollen membranes have to be estimated carefully, by accounting for the effects of the frame of reference and thermodynamic non-idealities. Not taking into account these effects, hampers the possibility of developing structure-property correlations to be used for the rational design of OSN membranes.

The most important recent advances in OSN are the introduction of polymers and hybrid materials with improved permeability, selectivity, and long term stability. Polybenzimidazoles, PIMs, block copolymers with hard and soft segments, hybrid materials containing MOFs and ZIFs and membranes based on pre-assembled nanoparticles appear as the best candidates for future developments.

The most challenging application in the next decades will likely be the separation of isomers. Solving this problem will open a new era in chemical, petrochemical, food, and pharmaceutical industry. Joined efforts from polymer chemistry (to synthesize and modify materials) and physical chemistry (to understand and define optimal transport properties) are necessary to make such new era a reality.

## Author contributions

MG conceived and wrote the manuscript. KB contributed to select the contents, prepared the figures and run calculations.

### Conflict of interest statement

The authors declare that the research was conducted in the absence of any commercial or financial relationships that could be construed as a potential conflict of interest.
